# Precision prevention in occupational health: a conceptual analysis and development of a unified understanding and an integrative framework

**DOI:** 10.3389/fpubh.2024.1444521

**Published:** 2024-09-18

**Authors:** Filip Mess, Simon Blaschke, Doris Gebhard, Julian Friedrich

**Affiliations:** Department Health and Sport Sciences, TUM School of Medicine and Health, Technical University of Munich, Munich, Germany

**Keywords:** precision health, workplace health promotion, worksite, employee, tailoring, occupational health and safety management, employee assistance programs

## Abstract

**Introduction:**

Precision prevention implements highly precise, tailored health interventions for individuals by directly addressing personal and environmental determinants of health. However, precision prevention does not yet appear to be fully established in occupational health. There are numerous understandings and conceptual approaches, but these have not yet been systematically presented or synthesized. Therefore, this conceptual analysis aims to propose a unified understanding and develop an integrative conceptual framework for precision prevention in occupational health.

**Methods:**

Firstly, to systematically present definitions and frameworks of precision prevention in occupational health, six international databases were searched for studies published between January 2010 and January 2024 that used the term precision prevention or its synonyms in the context of occupational health. Secondly, a qualitative content analysis was conducted to analyze the existing definitions and propose a unified understanding. Thirdly, based on the identified frameworks, a multi-stage exploratory development process was applied to develop and propose an integrative conceptual framework for precision prevention in occupational health.

**Results:**

After screening 3,681 articles, 154 publications were reviewed, wherein 29 definitions of precision prevention and 64 different frameworks were found, which can be summarized in eight higher-order categories. The qualitative content analysis revealed seven themes and illustrated many different wordings. The proposed unified understanding of precision prevention in occupational health takes up the identified themes. It includes, among other things, a contrast to a “one-size-fits-all approach” with a risk- and resource-oriented data collection and innovative data analytics with profiling to provide and improve tailored interventions. The developed and proposed integrative conceptual framework comprises three overarching stages: (1) data generation, (2) data management lifecycle and (3) interventions (development, implementation and adaptation).

**Discussion:**

Although there are already numerous studies on precision prevention in occupational health, this conceptual analysis offers, for the first time, a proposal for a unified understanding and an integrative conceptual framework. However, the proposed unified understanding and the developed integrative conceptual framework should only be seen as an initial proposal that should be critically discussed and further developed to expand and strengthen both research on precision prevention in occupational health and its practical application in the workplace.

## 1 Introduction

Precision prevention promises highly accurate, tailored health interventions for individuals and, potentially, populations (1). In this respect, precision prevention claims to directly target genetic, biological, behavioral, social and environmental determinants of health (2, 3) and to optimize non-pharmaceutical interventions based on these factors (4, 5). This ensures that the right support is provided to the right individual at the right time (2, 3, 6–10). Precision prevention thus extends the precision medicine approach by focusing on multiple determinants of health (6). In addition, precision prevention takes a holistic view of health according to the World Health Organization’s (WHO) biopsychosocial model of health, according to which not only biological but also psychological and social factors must be taken into account when considering people’s health (11). Beyond this, precision prevention also takes a lifespan perspective on health into account and, therefore, includes interventions across the entire lifespan (3, 12). Precision prevention aims to develop an expanded precision perspective and transfer knowledge to public health and health promotion research, focusing on healthy populations. Precision prevention may be viable within the whole prevention paradigm—primary, secondary and tertiary prevention (13). In summary, precision prevention goes beyond the genetic and clinical characteristics (personal omics profile) emphasized in precision medicine and encompasses behavioral, psychological, social and environmental contexts. This distinguishes precision prevention conceptually from precision medicine and a universal approach in the sense of “one size fits all” (14).

The relevance of precision prevention lies—similar to personalized treatment within the realm of precision medicine—among other things, in the fact that precision prevention enables a more effective approach to reaching individuals for preventive measures and health promotion, which thereby positively influences their health behaviors. Thus, their health can be better promoted. Precision prevention interventions are expected to achieve greater health impacts, such as greater effectiveness (14). Innovative person-centered concepts and approaches are needed to address individuals across their lifespan stages and in different settings, i.e., their most important living and working environments (15, 16).

### 1.1 Precision prevention in occupational health

The workplace is a particularly important setting for precision prevention, as working adults spend an estimated 30 to 40 percent of their waking hours at work (17, 18), and thus also for the implementation and delivery of health-promoting programs or occupational health in general (19–21). In addition, the workplace provides an efficient structure to reach large groups for health-promoting interventions and uses natural social networks (22). For example, workplace health services, which are part of the work organization, can be used with their structures and expertise to develop and implement workplace health promotion programs (23). Therefore, workplaces are an ideal setting for developing and implementing health promotion interventions as they offer access to a large number of adults and the opportunity to implement multi-level interventions targeting individual, organizational and environmental determinants of health and health behaviors (24).

The current state of research on precision prevention in occupational health can be described from different perspectives and scientific disciplines due to its interdisciplinary approach. This is presumably also a reason why—except the review by Mess et al. (25)—precision prevention in the occupational context has not yet been considered comprehensively or only concerning specific health conditions (26). The following two approaches describe publications on precision prevention in occupational health: (1) through general publications on precision prevention and (2) through specific publications on precision prevention in occupational health.

1. General publications and reviews on precision prevention provide initial access to the state of research, in some cases with initial references to occupational health. For example, Viana et al. (12) identified three studies (3/225; 1%) in their scoping review conducted in the workplace. Using the same search strategy, Mauch et al. (27) identified one additional study in the workplace setting in their review of precision prevention in the context of behavior change interventions. While there are many other reviews in the area of precision prevention, e.g., focusing on children (28, 29), patients in health care/nursing (30, 31), geriatrics (32), biomedical facets and -omics of precision prevention (33, 34), specific health behaviors, e.g., dietary behavior (29, 35), specific diseases, e.g., autoimmune diseases (36), diabetes (37, 38), obesity (29, 39), Alzheimer’s disease (40) or specific data analytics methods, e.g., machine learning (41), they do not mention the workplace as a central setting or focus on the employees.
2. In the context of precision prevention in occupational health, Mess et al. (25) showed in their review, after screening 3,249 articles, that there are 129 studies on precision prevention research in the field of occupational health. According to the cyclical model of Gambhir (42), with the four key stages of *risk assessment*, *customized monitoring*, *data analytics* and *interventions*, almost three-quarters of the studies addressed an *intervention* (74%). Only 14 percent of the articles focused on *risk assessment* and *customized monitoring* and 12 percent mainly included *data analytics* studies. Most of the involved studies focused on behavioral outcomes (e.g., physical activity/sitting behavior, eating habits, smoking/alcohol consumption; 38%), followed by psychological (e.g., mental health, stress, strain or depression; 23%) and physiological (e.g., musculoskeletal complaints or obesity; 19%) health outcomes (multiple mentioning was possible). The analysis of the study designs showed that randomized controlled trials (RCTs) were used in more than a third of all studies (39%), followed by cross-sectional studies (18%). In contrast, newer designs, such as just-in-time adaptive interventions (JITAIs), were rarely implemented. In addition to numerous other results, Mess et al. (25) presented the most important data analyses of all studies in their review. Regression analyses (e.g., 44% variance analyses or linear mixed models) were used most frequently. In contrast, machine learning methods (e.g., algorithms, Markov models) were only used in 8 % of the articles. Moe-Byrne et al. (26) conducted a systematic review of RCTs to assess the effect of tailored digital health interventions in the workplace. The interventions primarily aimed to improve employees’ physical and mental health, presenteeism and absenteeism. The studies showed positive effects of tailored digital interventions on presenteeism, sleep, stress levels and physical symptoms related to somatization. Although the digital interventions included in the review did not reduce anxiety and depression in the general working population, they significantly reduced depression and anxiety in employees with higher levels of psychological distress (26). In addition, Moe-Byrne et al. (26) reported in their review that most of the studies showed no improvement in absenteeism but a faster return to work among long-term sick workers who had received a tailored intervention.

Although the scoping review on precision prevention in occupational health by Mess et al. (25) systematically presented the identified studies and provided an initial insight, the authors identified several research gaps that must be addressed. These research gaps resulted from numerous other publications, particularly the 129 studies in the review (25).

This includes, among other things, the presentation of a unified understanding of precision prevention in occupational health, which still needs improvement due to the numerous, sometimes very different, definitional descriptions. For example, in their scoping review protocol, Ryan et al. (43) used several synonyms of the term precision (e.g., personalized, individualized, stratified, tailored) in combination with the term health as a search term. This broad concept and term of precision health was also used in some other publications (3, 27). In contrast, Mess et al. (25) decided in their scoping review to rely on the terminological description of Bíró et al. (13), using the term precision prevention and its associated definitions.

These selected terminological descriptions show that very different terms are currently used, depending on the scientific discipline and the underlying understanding of precision prevention (health, medicine) used [c.f., (44)]. Although the first very general descriptions of precision medicine and precision prevention have been proposed in some publications (13, 14, 45) and there are also initial setting-specific understandings of precision prevention [e.g., in healthcare (32), in pediatrics (28)], the workplace setting appears to have been neglected to date. Future research should, therefore, initially focus on systematically presenting and analyzing the terms and concepts of precision prevention used in occupational health (25). From this, a unified understanding of precision prevention in occupational health should be developed and proposed to apply the currently very heterogeneous understanding more uniformly in future research.

A further challenge in research on precision prevention in general and specifically in occupational health is that although some authors base their studies on frameworks (models, approaches, theories, etc.), the frameworks published to date are, at first glance, heterogeneous and include numerous, very different approaches. For example, the tiered model proposed by Gambhir (42), which has four stages: 1) *risk assessment*, 2) *data monitoring*, 3) *data analysis* and 4) *interventions*, is a promising approach. For example, in their scoping review, Viana et al. (12) rely on this stage model of precision prevention and use this structure to show that the aspects of precision prevention can be implemented in different stages. At the same time, the authors use the stages in their review for a structured presentation of the included studies, among other things. In their scoping review, Mess et al. (25) also rely on the general stage model (42) when presenting their results in occupational health. However, this stage model seems to be only one of several used in studies on precision prevention in general (46) and occupational health in particular.

Apart from the lack of a systematic overview of the frameworks used in precision prevention in occupational health to the best of our knowledge, no integrative conceptual framework has yet been developed in this field of research. In this respect, no framework currently appears to combine the essential aspects of precision prevention approaches and apply them to occupational health (25). Such an integrative conceptual framework could help scientists in occupational health research and those involved in occupational practice to understand precision prevention in the workplace in a setting-specific and holistic manner. In the future, such an integrative conceptual framework could help scientists as an initial orientation and system for occupational health research to test, further develop or specify it in their studies. However, such an integrative conceptual framework could also help those involved in occupational practice to promote the individual health of a usually very heterogeneous workforce in the company in an even more targeted manner.

### 1.2 Research gaps and objectives

In brief, the following research gaps in precision prevention in occupational health research can currently be identified based on the explanations above:

- The use and interpretation of the terms precision prevention and its synonyms are heterogeneous and have not yet been systematically presented and analyzed in occupational health.
- A unified understanding of precision prevention in occupational health has not yet been proposed for research and practice.
- A systematic presentation and analysis of frameworks used in occupational health to implement precision prevention aspects do not yet exist.
- An integrative conceptual framework for precision prevention in occupational health that combines various aspects of precision prevention approaches and applies them in occupational health has not yet been developed and proposed.

Therefore, this conceptual analysis aims to:

1. Systematically analyze the terms and concepts of precision prevention currently used in occupational health research to propose a unified understanding of precision prevention in occupational health.
2. Systematically present the frameworks for precision prevention in occupational health research and develop and propose an integrative conceptual framework for precision prevention in occupational health.

## 2 Materials and methods

This conceptual analysis’s methodological approach was mainly based on the steps of the key elements and stages from Hulland (47) and Jaakkola (48). In the first step, based on a literature search (25), we systematically integrated and synthesized the current research on precision prevention in occupational health (Sections 2.1 and 2.2). We followed the reporting guidance provided by the Preferred Reporting Items for Systematic Reviews and Meta-Analyses Extension for Scoping Reviews (PRISMA-ScR).

Based on this systematic approach, we used a mixed-methods design with qualitative and quantitative analyses to achieve a thorough and balanced understanding of precision prevention in occupational health. The qualitative content analysis, according to Mayring (49) (Section 2.3), was conducted to explore and synthesize the diverse definitions and descriptions of precision prevention in occupational health. We identified patterns, themes, and underlying concepts across the different publications by analyzing the terminology and contextual nuances. We then developed and proposed a unified understanding of precision prevention in the second step (objective 1). The third step used the systematic approach to quantitatively present the current frameworks (Section 2.3). It is joint or even a central element of conceptual articles [e.g., (47, 48, 50, 51)] that new theories, integrative/conceptual frameworks, models, phenomena, etc., are developed and presented based on analyses conducted and thus the presentation of existing knowledge. Qualitative analysis provided depth by delving into the meanings and nuances of precision prevention, while quantitative analysis provided breadth by giving a numerical overview of the used frameworks. Together, they offered a comprehensive picture that neither approach could achieve alone.

We then broadened the focus with both results and developed an integrative conceptual framework for precision prevention in occupational health (objective 2). Finally, based on conceptual review articles (47, 48), we identified numerous starting points for further development and future research.

### 2.1 Information sources, search strategy and eligibility criteria

The final original database search was carried out on January 4, 2024, including publications from six electronic databases (Web of Science^™^, Scopus^®^, Embase^®^, Ovid MEDLINE^®^, PubMed^®^ and APA PsycInfo^®^). Therefore, with the onset of the term “precision prevention” in 2010 (43), the period was from January 2010 to January 2024. The search string for titles and abstracts included the first focus on “precision” with the different terms “personalised/personalized,” “individualised/individualized,” “stratified,” “tailored” and “targeted” combined by the Boolean operator “OR.” The second focus was on the workplace-related terms “worksite,” “organisational/organizational,” “occupational,” “worker,” “employee” or “corporate.” Then, the terms of the two foci were combined by the Boolean operator “AND.” Database-specific proximity operators (i.e., “NEAR/x operator,” “PRE/x operator,” or “adj”) helped to find term combinations (e.g., “tailored intervention”). The focus groups were adults in working contexts, human tissue samples (e.g., genetic material) or historical datasets (e.g., health records) of workers. We focused on peer-reviewed publications (written in English) in primary empirical research studies (e.g., RCTs for intervention studies or cross-sectional studies for observational studies), study protocols (to capture planned studies) and conference proceedings. We excluded reviews and meta-analyses due to the possibility of secondary studies being potentially considered twice. Furthermore, we excluded grey literature, editorial articles, book chapters, dissertations, abstracts and posters due to the lack of a systematic peer review process and unavailable articles. For search string and eligibility criteria, see Supplementary Table S1.

### 2.2 Screening and data extraction

**Figure 1:**
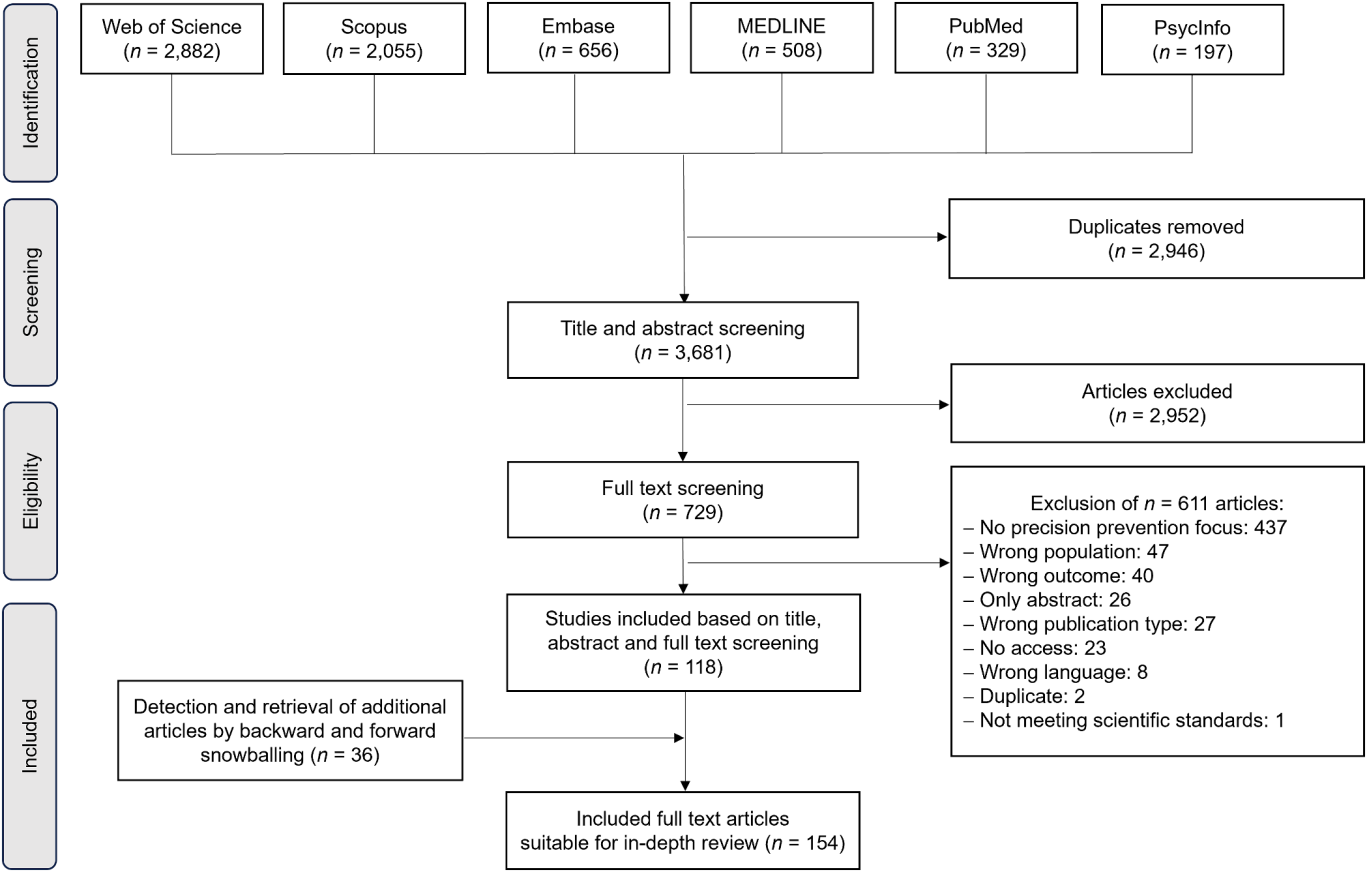
PRISMA-ScR flow chart for the procedure.

After retrieving and exporting all articles from the databases into the reference manager software EndNote^™^ (Alfasoft GmbH, Germany), the citations were transferred to the collaboration platform for systematic reviews “Rayyan App”^1^ (Rayyan Systems, Boston, Massachusetts, United States). We performed article screening in six combinations of two authors, resulting in a title and abstract screening of 50 percent for each collaborator. After all reviewers made independent judgments, the screening results of the reviewer pairs were merged and disagreements were resolved in discussions. A PRISMA-ScR flow chart for the procedure can be found in Figure 1.

This conceptual analysis focused on understandings and frameworks. In the next step, two reviewers searched all included articles (see Supplementary Table S2) for definitions or descriptions related to precision prevention. Whenever there were indications of an extended understanding of precision prevention, we transferred the corresponding paragraph to a spreadsheet using Excel^®^ software (Microsoft Cooperation, Redmond, Washington, United States) and included the text as material. We followed the same procedure for the used frameworks and concentrated the data on the model names (see Supplementary Table S3).

### 2.3 Data analysis and synthesis

Qualitative analyses were utilized to find coherence in the definitions or descriptions used to provide a unified understanding of precision prevention in occupational health. We used qualitative content analysis (49) and the software MAXQDA 2024 (VERBI Software). An inductive procedure was applied to identify the elements currently used in the literature to describe/define (aspects of) precision prevention approaches. An inductive procedure uses a bottom-up approach to analyze the data. It is an exploratory process where patterns, themes or categories emerge directly from the data instead of starting with preconceived categories. The essence of each segment was captured by identifying meaningful segments and assigning initial codes to them (49). The entire body of the selected text sequences represents the unit of analysis. Any phrase that refers to descriptive or definitional elements of (aspects of) precision prevention approaches was defined as a selection criterion. Each meaningful element, including individual words, formed the coding unit. The coding unit is the smallest segment representing a discrete piece of information that can be categorized. The selected text sequence of an article formed the context unit. A context unit is a larger segment of text that includes enough surrounding text to ensure that the meaning of the coding unit is clear and accurately understood (49).

The first step was the inductive extraction of the descriptive and definitional themes by summarizing. Two independent coders processed all the material to identify the themes by consensus. The content-related structuring technique was applied to the material following the identified themes in the next step. Subsequently, main categories and subcategories were developed inductively by summarizing the thematically structured material within the specific themes. The category system and the coding agenda were steadily revised and complemented by two coders. A reliability proof of the final category system was made for the entire material. Disagreement between the two coders was resolved by discussion and consensus. Finally, the qualitative data was quantified by calculating frequencies.

In quantitative analyses, the number of each framework was counted. Moreover, the frameworks were assigned to different higher-order categories according to previous literature [e.g., (52)] and discussed by two reviewers.

## 3 Results

Of the *n* = 154 articles screened, 19 percent included descriptive elements for (aspects of) precision prevention approaches. These identified *n* = 29 articles, thus representing the material for the qualitative content analysis of definitions or descriptions. Furthermore, *n* = 49 articles named or referenced at least one framework. Multiple mentions lead to 64 frameworks for quantitative analyses.

### 3.1 Qualitative results: understandings and definitions

The inductive procedure revealed seven themes currently used as descriptive elements for (aspects of) precision prevention approaches and could thus serve as a basis for developing a unified understanding of precision prevention. Across the seven themes, 199 quotations were coded within 32 main categories. Each theme was individually described and the presentation sequence moved from general to more specific aspects.

#### 3.1.1 Theme 1: different wordings (*n* = 43)

Across all documents, ten different wordings for (aspects of) precision prevention approaches were identified. With 17 mentions, the word tailored was used most frequently. The umbrella term precision prevention (13) was only mentioned in one document. Table 1 presents the different wordings sorted by frequency.

**Table 1:** Frequencies of different terminologies.

Terminologies	Frequencies (*n*)	Percentages (%)
Tailored	17	40
Personalized	9	21
Individualized	5	12
Person-centered	4	9
Adaptive	2	5
Specific	2	5
Targeted	1	2
Customized	1	2
Addressee orientation	1	2
Precision prevention	1	2
Total	43	100

#### 3.1.2 Theme 2: describing by contrasting (*n* = 14)

Fourteen quotations from nine authors described the precision prevention approach by distinguishing it from others. The most important differentiator was the size of the population whose data was used or to whom the interventions were directed: It was differentiated whether the focus was on the entire population, on a specific group or the individual. Two quotations explained that precision prevention (1) focuses not on the entire population but on groups that share common characteristics and thus form a specific profile or cluster (53, 54). Five quotations set the precision prevention approach (2) apart from group-centered approaches by placing the individual at the center of interest (53, 55–58).

In addition, the precision prevention approach was contrasted with a “one-size-fits-all approach” in four quotations (53, 57, 59, 60) and it goes beyond the original approaches to promoting healthy behavior (61). Furthermore, the precision prevention approach was also described in contrast to medicine. Thus, precision prevention generally focuses on the person and not the disease [in contrast to medicine (56)], and in occupational health, no biomarkers are used [in contrast to individualized medicine (61)].

#### 3.1.3 Theme 3: aims and benefits (*n* = 26)

Statements about the aim or benefit of (aspects of) precision prevention approaches are presented in 19 documents, whereby two different aspects of meaning could be coded in seven of them. Four main categories were developed inductively. The improvement of interventions (in four different aspects) was most frequently mentioned as an aim/benefit of precision prevention approaches. Table 2 shows the main and sub-categories and their frequency of mention.

**Table 2:** Aims and benefits of precision prevention approaches.

Aims and benefits	Frequencies (*n*)	Percentages (%)
Improve interventions		
Increase effectiveness	5	19
Improve implementation	3	12
Increase (economic) efficacy	2	8
Address individual needs and requirements	1	4
Improve the relevance and availability of health information	5	19
Improve professional knowledge and practice	5	19
Identify/classify relevant individuals/groups	5	19
Total	26	100

#### 3.1.4 Theme 4: used variables (*n* = 48)

**Figure 2:**
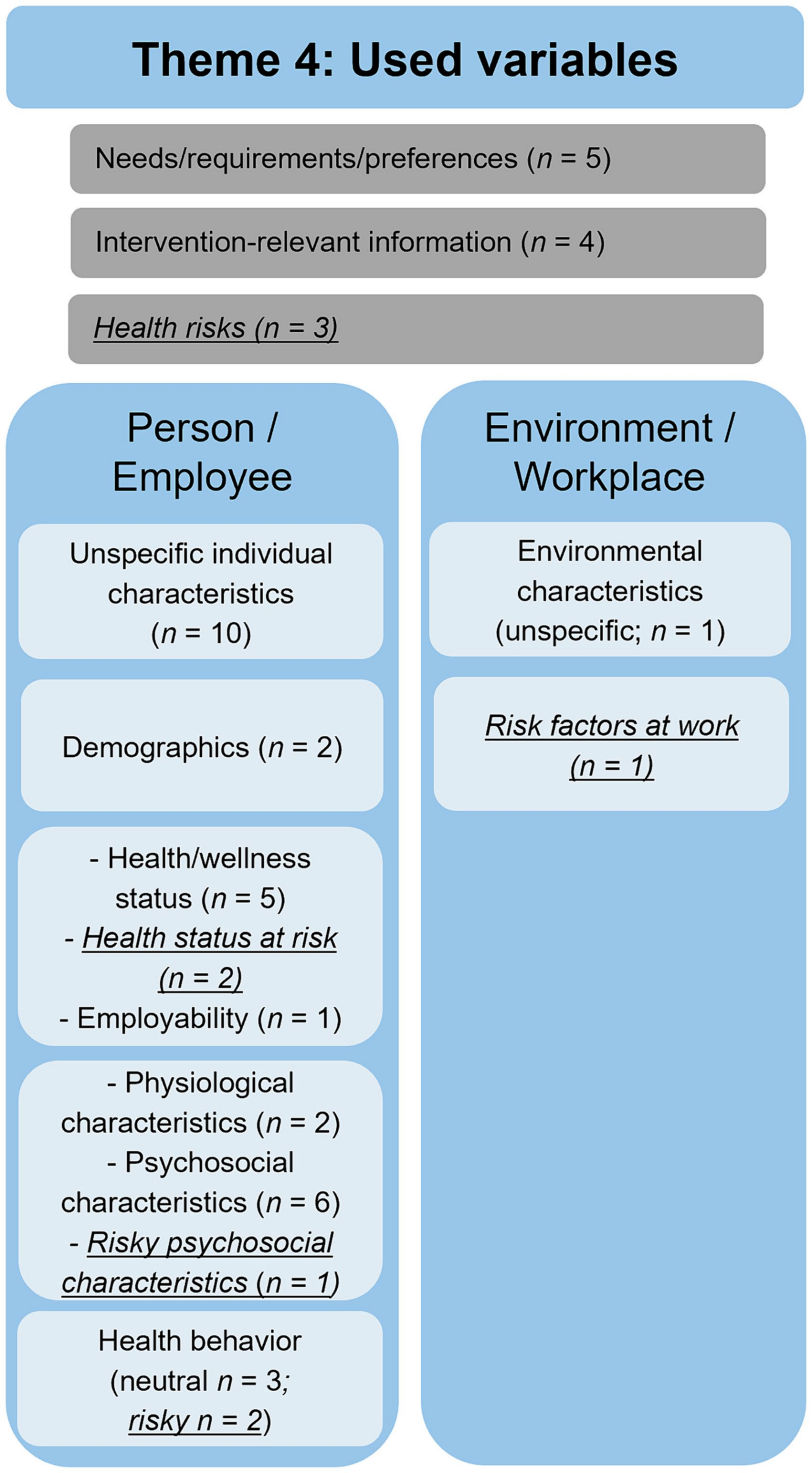
Overview of the used variables. Underlined topics refer to a risk-oriented approach.

With 48 mentions in 21 documents, the theme “used variables” was the most prominent and frequently mentioned topic across all documents. Within this theme, three main categories were inductively developed (person-/employee-related variables: *n* = 34; environment-/workplace-related variables: *n* = 2; and unspecific variables: *n* = 12). The categories mainly contained variables mentioned in the context of assessments and addressed by interventions. Most variables were formulated neutrally within the subcategories and not defined as risks or resources. Nevertheless, a pathogenetic perspective was predominant, as nine variables were associated with a health risk, but no variable was associated with a health resource (see Figure 2).

#### 3.1.5 Theme 5: data analytics (*n* = 11)

Data analytics was mentioned eleven times in ten documents, inductively grouped into four content categories. The identification of profiles (*n* = 5) was most frequently associated with data analytics, for example, with psychological profiles (59) or risk profiles (62). Pre-defined algorithms (*n* = 3), for example, if-then algorithms select relevant feedback (63) or generate tailored content for the users (64). Machine learning (*n* = 2) and big data approaches (*n* = 1) were mentioned less frequently, but machine learning techniques can, for example, generate an individual-level predictive model based on many predictors (58, 65).

#### 3.1.6 Theme 6: provided aspects (*n* = 36)

All in all, 19 documents delivered descriptive elements of what is provided in a precision prevention approach. The inductive category development resulted in four main categories (intervention: *n* = 18; feedback: *n* = 9; counseling: *n* = 2; and unspecific: *n* = 7), including 36 quotations.

Within the subcategories of interventions, direct treatment was mentioned most frequently (*n* = 13): most interventions were behavioral treatments, for example, behavior change interventions (66–68) or tailored to work stress and performance (69). Another category, “unspecific treatment,” contained again interventions that were not described or explained further [e.g., “provide a suite of support tools” or “risk-reduction activities” (70)]. In the category of no direct treatment, mainly information on behavior change (*n* = 3; e.g., “information that allows translating knowledge to action” (70); “offering opportunities for behavior change and improvement” (71) or information and suggestions for a treatment [*n* = 2; “suggestions to reduce those risks” (70); “advice on whether or not to take consecutive actions” (62)] were given.

The feedback category comprised permanent feedback on the health status [*n* = 2; e.g., monitoring with direct biofeedback and visualization of health data (66)] and feedback before an intervention (*n* = 7). The current employability and health status (62), lifestyle habits (71) or knowledge of health risks (70) were related to the health status of the employees before an intervention. Furthermore, other unspecific feedback was provided [e.g., “relevant feedback messages from a database” (63); “employee receives feedback on the results from the assessments by a consultant” (62)].

The counseling sessions were before and/or after an intervention (*n* = 1 each). Individual coaching was based on a broad assessment followed by a tailored intervention (62). Counseling was based on intervention results (66). The unspecific category included information or messages that were not described or explained further [e.g., “information that is tailored” (72) or “creation of unique messages” (57)].

#### 3.1.7 Theme 7: mode of delivery (*n* = 21)

Across all documents, two delivery modes (73) of precision prevention approaches could be identified: electronic and human interactional modes of delivery. Electronic delivery was the dominant mode, with 19 mentions, including, for example, services via websites (70, 72), health dashboards (66, 70), apps (66, 71) and mobile health approaches or systems (68, 74). From the context of the quotations, it could be assumed that electronic also means automated, i.e., everything offered digitally is created automatically without direct human involvement. The two mentions of human interactional mode of delivery referred to counseling activities before and/or after an intervention.

### 3.2 Frameworks and quantitative analysis

The quantitative analysis investigated *n* = 64 frameworks (models, approaches, theories, etc.). The different directions ranged from general psychological models [e.g., *Social Cognitive Theory* (75)] to articles that approach a particular topic or niche [e.g., *5C Psychological Antecedents of Vaccination* (76)]. Therefore, we categorized the frameworks (see Table 3) into eight higher-order categories.

**Table 3:** Frequencies of articles referencing categories of frameworks (*n* = 64).

Higher-order categories and frameworks	Frequencies (*n*)	Percentages (%)
Intervention design and evaluation models	14	23
Intervention mapping SWOT analysis RE-AIM framework PRECEDE-PROCEED model Diffusion of Innovation theory Participatory action research Intervention development framework 4 stages model	5 2 2 1 1 1 1 1	
Decision-making theories	10	16
Transtheoretical model of health behavior change Elaboration likelihood model of persuasion 5C psychological antecedents of vaccination Stages of change approach	5 2 2 1	
Socio-cognitive theories	10	16
Social cognitive theory Health action process approach Social learning and cognitive-behavioral models Model of social action theory Self-regulation theory	5 2 1 1 1	
Belief-attitude theories	7	11
Theory of planned behavior Health belief model Integrative model of behavioral prediction	5 1 1	
Person-centered approaches	7	11
Person-centered model Targeted and tailored approaches Personalized classification model Person-centered-practice framework	4 1 1 1	
Workplace health models	6*	10
Job demand-control-(support) model Job demands-resources model Effort-reward imbalance model Total worker health Demand-induced strain compensation recovery model	3 2 1 1 1	
Precision Health/Medicine approaches	4	7
Precision medicine Precision health model P4 medicine framework	2 1 1	
Stress-coping-well-being theories	3*	5
Burnout cascade model Reserve capacity model Conservation of resources theory Two continua model for mental illness and mental health	1 1 1 1	

*Due to multiple mentions, an article may deal with several frameworks of the same category so that the individual frameworks do not add to the sum of the category. SWOT, strengths, weaknesses, opportunities and threats; RE-AIM, reach, effectiveness, adoption, implementation, maintenance; PRECEDE, predisposing, reinforcing and enabling constructs in educational diagnosis and evaluation; PROCEED, policy, regulatory and organizational constructs in educational and environmental development; 4 Stages Model = (1) Evaluation of ground setting, (2) basic intervention, (3) progressive intervention, (4) follow up-stage; 5C, confidence, complacency, constraints, calculation, collective responsibility; P4, predictive, preventive, personalized, participatory.

#### 3.2.1 Precision health/medicine and person-centered approaches

Our analyses focused on the referenced models categorized as *Precision Health/Medicine Approaches* (with four articles mentioning frameworks) and *Person-Centered Approaches*, with seven articles mentioning frameworks. *Precision Health/Medicine Approaches* focus on tailoring healthcare interventions and treatments to individuals or subpopulations based on their genetic, environmental, and lifestyle factors. The *Precision Health Model* (42, 77), *Precision Medicine* (78, 79) and the *P4 Medicine Framework* emphasize the integration of advanced technologies and comprehensive data analysis to enable personalized and proactive healthcare strategies. Therefore, this category is close to the understanding of the current conceptual analysis. In the *Person-Centered Approaches* category, the individual’s needs, preferences and values in interventions are prioritized. These frameworks, including the *Person-Centered Model* (54), the *Targeted and Tailored Approaches* (55, 80), the *Personalized Classification Model* (81) and the *Person-Centered-Practice Framework* (82–84), emphasize the importance of understanding and incorporating the unique characteristics and circumstances of each person to enhance the effectiveness and relevance of interventions.

#### 3.2.2 Intervention design and evaluation models

Most articles (*n* = 14) mentioned frameworks in the *Intervention Design and Evaluation Models* category. In this category, most of the articles (*n* = 5) referenced intervention mapping (85, 86), followed by *SWOT Analysis* (short for strengths, weaknesses, opportunities, threats); credited to Stewart et al. (87) and the *RE-AIM Framework* (Reach, Effectiveness, Adoption, Implementation and Maintenance) by Glasgow et al. (88) with two mentions each. These models and theories are all public health and organizational development frameworks to guide interventions’ planning, implementation, and evaluation. They emphasize a systematic approach to understanding, addressing, and evaluating complex issues through stages, assessments, and participatory methods to maximize effectiveness and sustainability.

#### 3.2.3 Workplace health models and stress-coping-well-being theories

Furthermore, the category *Workplace Health Models* was mentioned in six articles. The *Job Demand-Control-(Support) Model*, the *Job Demands-Resources Model* (89) and the *Effort-Reward Imbalance Model* are often cited models that state the interplay between occupational factors and employee well-being, highlighting the importance of balancing demands, control and resources to mitigate negative health outcomes and promote occupational health. *Stress-Coping-Well-Being Theories* were less mentioned (*n* = 3). These frameworks have a broader conceptualization than *Workplace Health Models*, understanding how stressors, coping mechanisms and individual resources interact to influence well-being outcomes in general.

#### 3.2.4 Decision-making, socio-cognitive and belief-attitude theories

Furthermore, in the categories *Decision-Making Theories* (*n* = 10), *Socio-Cognitive Theories* (*n* = 10) and *Belief-Attitude Theories* (*n* = 7), frameworks reference processes and factors that influence how individuals make health(y) choices and give an understanding of how individuals’ beliefs, perceptions and social environments influence their behaviors and decision-making processes. Moreover, individual beliefs, attitudes and perceptions towards a behavior, coupled with perceived social norms and control over the behavior, influence behavioral intentions and subsequent actions. These theories highlight the significance of understanding individuals’ cognitive processes, beliefs about the behavior’s outcomes and perceived barriers and facilitators in predicting and explaining behavior change.

## 4 Discussion

The first objective of this conceptual analysis was to analyze the terms and concepts of precision prevention in occupational health using qualitative content analysis and propose a unified understanding. The second objective was to systematically present the frameworks of precision prevention currently used in occupational health research to develop and propose an integrative conceptual framework for precision prevention in occupational health. Given these different objectives, the results of the qualitative content analysis of the definitions or descriptions are first discussed and a proposal for a unified understanding of precision prevention will be given (Section 4.1). Secondly, the results of the quantitative analyses of the frameworks are discussed (Section 4.2). Thirdly, based on some selected and discussed frameworks (Section 4.1), the proposed integrative conceptual framework is finally described (Section 4.3) and future options for further development are outlined (Section 4.4).

### 4.1 Qualitative content analysis of definitions and proposal of a unified understanding

The qualitative analysis of the definitions or descriptions of precision prevention in occupational health revealed that 29 of the 154 publications contained definitions or descriptions of precision prevention. Overall, there is a wide range of wordings. Thus, the accuracy of the construct and its quality are also heterogeneous. Bíró et al. (13) defined “precision prevention” as an umbrella term, while the most mentioned wording in our analysis was “tailored,” followed by “personalized” and “individualized.” These terms may be overrepresented because most of the studies were interventions and these were often called “tailored/personalized/individualized interventions.” When developing or analyzing an intervention, researchers may focus more on the tailored/personalized/individualized intervention than the conceptual background of precision prevention. These similarities are consistent with general descriptions of precision prevention (90, 91). Accordingly, precision prevention refers, on the one hand, to the grouping of individuals who are subdivided from a population into smaller groups. On the other hand, precision prevention also refers to developing and implementing targeted, i.e., precise, interventions (90, 91). This non-uniform, sometimes diffuse or contradictory understanding of precision prevention in occupational health can also be found in precision prevention in general (43) or even in precision medicine (92). On the one hand, the heterogeneity and the associated qualitative differences are partly because the research field of precision prevention is still very young and developing (12, 25, 43). This finding confirms again the development and discussion of a unified understanding. On the other hand, it is also because precision prevention is still strongly characterized by medical approaches. This was represented in the qualitative results, contrasting the precision prevention approach to precision medicine, e.g., using biomarkers. However, the focus was mainly on health risks and a pathogenetic perspective. Several authors criticize that too little attention is paid to social and behavioral science approaches and aspects of precision prevention (3, 44). The heterogeneity of the definitions or descriptions can also result from the different interests, competencies and skills of the people involved in occupational health, such as practitioners and researchers (93, 94). Therefore, other ways of thinking (12), such as a positive psychological view on resources and a holistic, interdisciplinary view, e.g., health sciences, have not yet taken place enough (25).

The articles’ most mentioned aims or benefits were improvements, either of interventions (implementation/efficacy/effectiveness) or the work of practitioners (availability of information or knowledge and practice). This aligns with using digital aids and developments such as big data analyses with, for example, machine learning and algorithms. However, the limited number of mentions in the “data analytics” compared to “used variables” indicates that while data collection and processing is important, it may not yet be fully integrated into practice. In many cases, the challenges are complex, so elaborate approaches could help to understand the full extent and obtain a holistic overview. To address the diverse aspects of different individuals, groups or both, it is necessary to identify or classify them, mainly by profiling and other elaborate analysis techniques. Furthermore, different layers of variables can be considered (95), i.e., the person (employee) and environment (workplace). In considering a holistic approach, environmental factors and workplace-related variables are also important but underrepresented in the analyzed articles. Science and practice may focus more on behavior than on circumstances. On the one hand, this means that environmental influences are not yet considered as relevant as behavioral influences and the field of precision environmental health with exposome research is still relatively young (96). On the other hand, interdisciplinary cooperation and exchange between occupational medicine, which focuses on the person and behavior and public health, which focuses on the environment and conditions, could be strengthened (97, 98).

Regarding “provided aspects” and the “mode of delivery,” electronic delivery emerged as the predominant approach, with services often provided through health dashboards, apps, and mobile health platforms (66, 68, 70, 71, 74). This reflects the increasing reliance on digital technologies to deliver person-centered care, offering convenience and accessibility. However, the limited mentions of a human interactional mode of delivery indicate a potential gap in providing emotional or psychological support, essential for a holistic approach to health and well-being. Tailored feedback regarding the individual health status is mostly given before an intervention. Nevertheless, a personal component and multiple feedback loops may be helpful to support and motivate the individual. Permanent feedback on the health status was given in two articles and feedback in the monitoring phase could be discussed in in-person counseling before proposing a tailored intervention (62). A second feedback loop could be after the intervention (66) regarding the actual and updated health status and counseling regarding behavioral change or adaptations in the workplace. This would lead to a holistic approach with multiple feedback components and an interplay between digital aids and personal human contact (99).

Due to the different analysis components and the identified themes, we propose a unified understanding of precision prevention in occupational health and present it for further discussion:

Precision prevention in occupational health is, in contrast to a “one-size-fits-all” approach, identifying and classifying individuals or groups with their needs and requirements in the work context. Data collection and analytics with personal and environmental factors focusing on risks and resources provide a precise data basis for characterizing the profiles of individuals or groups to guide interventions. A feedback component of this characterization for the individual or the group can be delivered digitally or personally. Mostly named tailored but also personalized, individualized or person-centered interventions in the work context are developed, implemented and adapted to increase the interventions’ implementation, efficacy and effectiveness.

### 4.2 Quantitative analysis of models, frameworks, and approaches

The range and diversity of the various frameworks (models, approaches, theories, etc.) used in occupational health is wide. One reason is that the publications focused on very different aspects of precision prevention. For example, some publications focused exclusively on developing, implementing and evaluating tailored interventions. *Intervention Design and Evaluation Models* were then selected accordingly. In other studies, the focus was, for example, on changing specific employee behaviors (physical activity, dietary behavior, etc.) to align health-promoting interventions precisely with the employees’ willingness to change (e.g., *Decision-Making Theories*). In contrast, models that refer specifically to the aspects of precision prevention (*Precision Health/Medicine Approaches*; *Person-Centered Approaches*) were rarely used in the publications identified. This is mainly because the research field of precision prevention is still in its infancy; therefore, stage models [e.g., Gambhir (42)] have only been developed and published in recent years.

However, the wide variety of frameworks used can also be explained by the fact that numerous players from very different scientific disciplines are active in the occupational setting and precision prevention [cf., (12, 100)]. For example, health or occupational psychologists tend to focus on the personal and psychological aspects of employees’ health (relating more to *Decision-Making Theories*, *Socio-Cognitive Theories*, and *Belief-Attitude Theories*) and thus on mental health. Occupational physicians tend to focus more on biological, medical and physical aspects of physical health (relating more to *Workplace Health Models*, e.g., the Job Demands-Resources Model). Health scientists tend to be responsible for occupational health management in general, planning workplace health promotion programs and implementing and evaluating specific interventions (relating more to *Intervention Design and Evaluation Models*). Due to this multitude of frameworks used in occupational health, several authors have recently developed or proposed integrative models/frameworks [e.g., (100–102)].

As a central aim of this conceptual analysis is to develop an integrative conceptual framework for precision prevention in occupational health, only those models that are conceptually close to such a framework (due to the focus on precision prevention or the occupational setting) and therefore provide precious knowledge for its development are discussed in detail below. In line with the recommendations for writing conceptual analyses (47, 48), the focus of the discussion is extended beyond the identified models and numerous further aspects are included.

#### 4.2.1 Precision health/medicine approaches and person-centered approaches

The frameworks presented and categorized illustrate great heterogeneity and reference to numerous facets of precision prevention and health (behavior). The models within these two categories (*Precision Health/Medicine Approaches* and *Person-Centered Approaches*) strongly connect and overlap with the frameworks (models, approaches, theories, etc.) used in general publications on precision prevention (health, medicine). For example, the aforementioned stage models (*Precision Health/Medicine Approaches*), such as the model by Gambhir (42), are similar to general precision prevention models, such as the model proposed by Conrad et al. (36). However, it does not take up the circular progression. Some of the general models on precision prevention focus on two of the three stages mentioned above, *assessment* and *data analytics* (103), only on *data analytics*, mainly with machine learning and/or artificial intelligence (AI) (41, 104, 105) or only on *interventions* (32). In addition, there are some general models with a stronger focus on identifying individuals in the sense of profiling/phenotyping (34, 104, 106–108). In precision medicine, models have been developed that focus on comprehensive risk assessment, with many bio-medical (especially -omics), behavioral, social and psychological determinants (109–111). Some models are strongly medically orientated and focus on the treatment of patients (112–114) or are targeted at specific diseases, e.g., depression (115) or type 1 diabetes (116). In addition, there are currently also models used in patient-centered care or healthcare that serve to better identify and treat patients with a high health risk (117–121). Finally, Payne et al. (122) have proposed a framework for a precision health system that includes aspects of precision medicine with public health. However, no model appears to have been developed for specific use in occupational health.

Despite these different focuses, priorities and approaches, the frameworks (models, approaches, theories, etc.) in the context of precision prevention/health/medicine have some things in common: On the one hand, they use innovative data analytics (e.g., AI and machine learning) to identify individuals or groups (profiles) based on a large amount of personal data to then offer them a tailored intervention (treatment). On the other hand, digital technologies are also often used to continuously monitor the health-related data of individuals or groups over time.

#### 4.2.2 Workplace health models

In addition to the *Workplace Health Models* already described (Section 3.2.3), numerous very general frameworks in workplace health promotion deal with employee health and, therefore, provide essential knowledge for developing the integrative conceptual framework for precision prevention in occupational health. For example, Lecours et al. (100) published an overview of concepts of integrative prevention at work in their concept analysis and meta-narrative review and described 20 different concepts. One promising model is the conceptual model for integrated approaches to protecting and promoting worker health and safety (102, 123). This conceptual model was developed to specify the causal pathways through which workplace policies, programs and practices are expected to influence worker health and safety outcomes. For this reason, the model encompasses the *characteristics of the worker/workforce and the enterprise* and considers the *workplace policies, programs and practices*. In the first step, these factors are linked to different *conditions of work* and *worker proximal outcomes*, and in the second step, to *worker and enterprise outcomes* (102). This model is of high complexity and encompasses many interrelationships across multiple dimensions. The strength of this model is that it represents diverse theoretical perspectives, including the social-ecological model, the social contextual model of health behavior change, the hierarchy of controls, organizational ergonomics, participatory frameworks, job strain, and the socio-technical system theory (102). However, this integrative model also overlaps with the models presented in Section 3.2 and the categories formed from them (e.g., *Socio-Cognitive Theories*, *Belief-Attitude Theories*). The integrative model also offers some options for expansion, as it does not, for example, have any antecedents, such as access to resources or motivation for the implementation of integrative prevention (100). Nevertheless, this integrative model has been used over 100 times in various studies since its publication and, therefore, appears to have established itself in the field of occupational health. However, after reviewing the citations, it was found that none of the studies that used the model of Sorensen et al. (102) included aspects of precision prevention.

These two model categories (*Precision Prevention/Health/Medicine Models*; *Workplace Health Models*) were developed for different intentions and purposes and are now used in specific studies. The *Precision Prevention/Health/Medicine Models* primarily outline the steps and possible approaches to prevention in the sense of a multi-stage or continuous process to prevent individuals’ diseases or promote their health based on various data (42). In contrast, *Workplace Health Models* primarily aim to describe the various influencing factors (determinants), their interrelationships and their effects on employees’ health in their working environment (102). Despite these differences, the two model categories appear to have similarities. They both attempt to comprehensively describe numerous factors (determinants) of individual health to prevent disease or promote health. In addition, both model categories also aim to identify relevant causes or starting points that can be used in intervention studies to promote individuals’ health or prevent disease. Due to these similarities and to bring together the special features of the respective models, an integrative conceptual framework will be developed and proposed in the following.

### 4.3 Development and proposal of an integrative conceptual framework for precision prevention in occupational health

Based on the recommendations for possible objectives and structuring of conceptual review papers (47, 48, 50, 124), and according to the objectives of this conceptual analysis, an integrative conceptual framework for precision prevention in occupational health was developed that combines (1) various frameworks of precision prevention currently used in occupational health research (Sections 3.2 and 4.2), with (2) aspects of general *Precision Health/Medicine* and *Person-Centered Approaches* (Sections 4.2.1 and 3) also with general *Workplace Health Models* (Section 4.2.2).

When developing the integrative conceptual framework, we favored a *conceptual framework* rather than a *conceptual model* for the following reasons. According to the explanations of Brady et al. (125), a conceptual framework can be understood as a group of broadly defined and systematically organized concepts. Accordingly, conceptual frameworks should provide a focus, a rationale and a visual representation of what can be studied (125, 126). In contrast, conceptual models are more concrete, propose relationships (or causal linkages) between a set of concepts and therefore, provide better guidance, e.g., for prevention and intervention measures (125).

**Figure 3:**
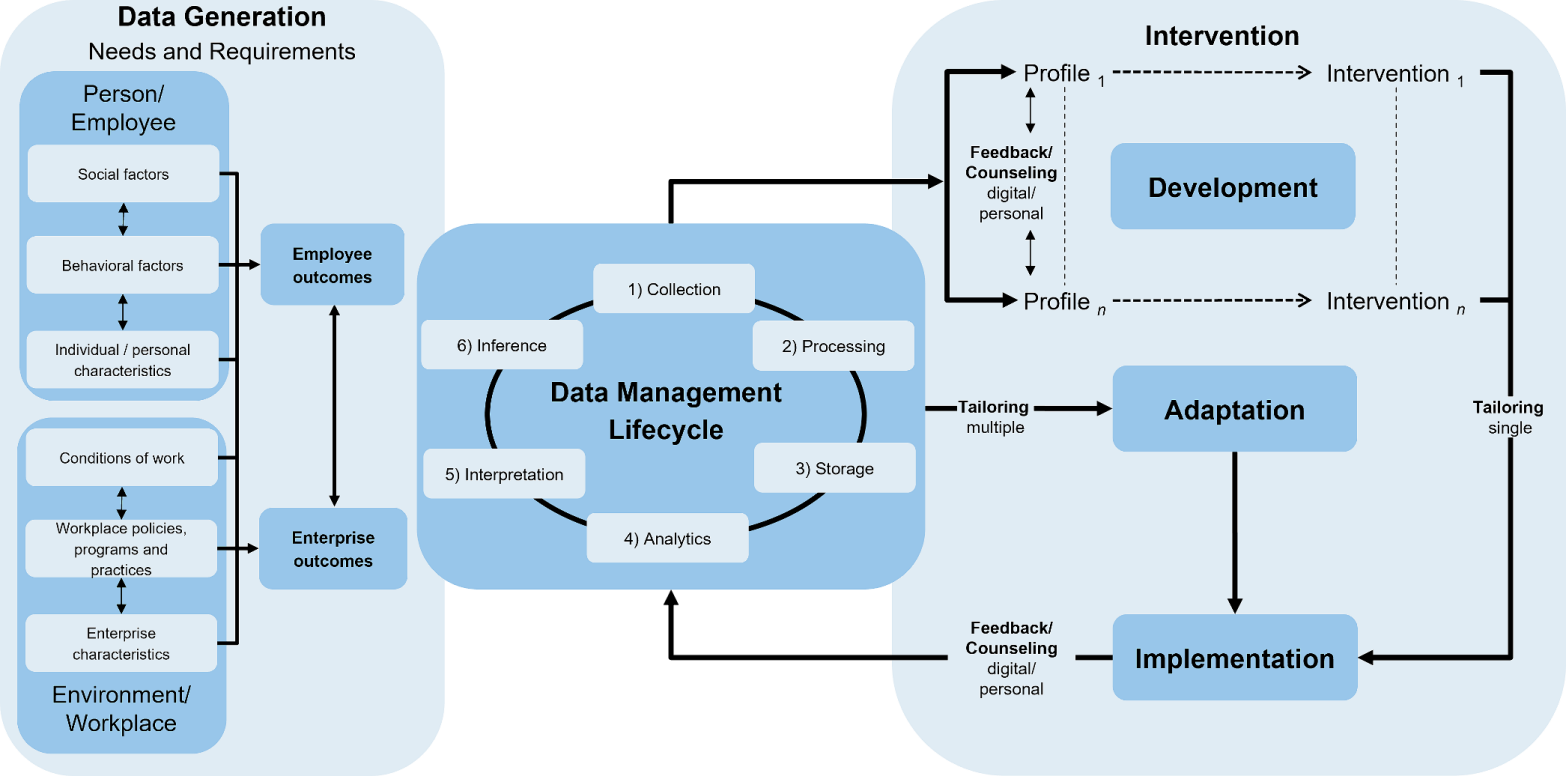
Integrative conceptual framework of precision prevention in occupational health.

The starting point of our considerations was to bring together the two central model categories described above (*Precision Health/Medicine Approaches*, *Person-Centered Approaches* and *Workplace Health Models*) in an integrative conceptual framework. Based on the temporal component in the stage models of precision prevention, our integrative conceptual framework (see Figure 3) also takes up this aspect. It proposes three consecutive and interlinked steps as an initial overarching structure: (1) *Data Generation* (of Needs and Requirements), (2) *Data Management Lifecycle* (collection, processing, storage, analytics, interpretation, inference) and (3) *Intervention* (development, implementation, adaptation). Due to this temporal structure, the integrative conceptual framework can be used as a process model in which the individual, interlinked stages can be run sequentially and recurrently (25). The needs and requirements presented in the *Data Generation* stage provide an overview of which data could potentially be collected to work with them in the *Data Management Lifecycle*.

The first stage, (1) *Data Generation*, primarily provides an overview of the various needs and requirements (determinants and outcomes) that could be generated and relate to both the individual (employee) and the organization (enterprise). Adapted from Glass et al. (127) and Brady et al. (128), the determinants can initially be categorized as *person/employee* and *environment/workplace*. The determinants of a *person/employee* are those that lie within the person, including individual/personal characteristics and behavioral and social factors. They can be described as a personal omics profile (13, 127). According to Martin et al. (129), the determinants of an *environment/workplace* can include aspects at the micro level [e.g., conditions of work (102)] as well as aspects at the meso- or macro-level (e.g., enterprise characteristics). In the vertical structure of the factors, the proposed framework also attempts to show that some factors are closer to the person/employee and, therefore, describe or influence them more directly. For example, the determinants of *conditions of work* usually have a more direct effect on the *person/employee* than general *enterprise characteristics*. As we have focused exclusively on the workplace setting, only those factors relevant to the integrative conceptual framework in this setting are presented here. However, other determinants, such as those presented in the model by Dahlgren et al. (95), affect employees’ health but have a greater influence outside the workplace setting, for example, general socioeconomic, cultural and environmental conditions.

Based on the theoretical preliminary work by Bíró et al. (13), the reviews by Viana et al. (12) and Mess et al. (25) and taking into account the findings from the identified *Precision Health/Medicine* and *Person-Centered Approaches* (34, 103, 110, 117) and *Workplace Health Models* (102, 130, 131), we propose the following factors/determinants for the integrative conceptual framework in the data generation stage:

- Individual/personal characteristics
- Behavioral factors
- Social factors
- Conditions of work
- Workplace policies, programs and practices
- Enterprise characteristics.

For the outcomes associated with the determinants, the employee can be considered on the one hand and the enterprise on the other. Based on the included studies and models on precision prevention (115, 116) and occupational health (102, 130, 132, 133), numerous outcomes, both for the employee and the enterprise, can be integrated into the integrative conceptual framework. For clarity reasons, the integrative conceptual framework includes only the two overarching aspects (employee and enterprise outcomes). However, these must be further concretized and differentiated when applying the framework in research and occupational practice.

Based on these possible determinants to be considered and their associated outcomes in the *Data Generation* stage, the *Data Management Lifecycle*, which has six steps (collection, processing, storage, analytics, interpretation, inference), should be run through. In the *Data Management Lifecycle* stage, it is important to decide how the data should be processed and where it should be stored to then identify profiles in the next step using various data analysis methods. Profiling is a method or process that aims to identify and characterize subgroups of individuals based on observable characteristics (e.g., health behavior or health) that can be attributed to a common set of underlying factors (134). If the results of the review by Mess et al. (25) are considered, most of the articles examined a profile approach with latent profile analysis or cluster analysis (11/16 studies; 69%), followed by machine learning methods (5/16 studies; 31%). This methodological approach is also evident when considering the data analysis methods used in general precision prevention models. Accordingly, profiling (34, 104, 106–108) and machine learning (41, 104, 105) are the most frequently used approaches. In this stage (*Data Management Lifecycle*), it is also important to interpret the results obtained and present them in an understandable way for practical application. Feedback should also be provided in this step, for example, in integrated health portals (42), health apps (71) or dashboards (66, 70).

Following the temporal logic of the framework, specific interventions should be developed and implemented for each identified profile (*Intervention*). The challenge here is to develop specific interventions that match the complex behavioral and health phenomena and then implement them (e.g., with implementation strategies and models) in real-world contexts (3, 6, 135). *The Intervention Design and Evaluation Models* mentioned in Section 3.2.2 (e.g., *RE-AIM Framework*, *Intervention Mapping*) can be used as an initial orientation for this and are particularly helpful for further differentiation and concretization. According to the distinctions made by Hekler et al. (3), interventions can be categorized into different types that exist on a continuum: generic, targeted, adaptive and continuous interventions. The review on precision prevention in occupational health (25) shows that many intervention studies differ greatly in study design, data analysis methods, etc. Overall, however, it can be seen that precise and learning interventions, such as adaptive or continuous tuning interventions, for example, JITAIs, micro randomized trials (MRTs) or *N*-of-1 trials, are currently rarely used in general precision prevention research (3, 6) and occupational settings (25).

In line with the above-mentioned cyclical framework, the development of the intervention is followed by its implementation in the occupational context. Depending on the development of the intervention (study and intervention design) and the possibilities for implementation in occupational practice, it may be possible to conduct multiple tailoring of the intervention in addition to a single tailoring. For this purpose, it is necessary to collect selected data from the individuals or groups involved (profiles) several times after implementing the intervention and thus adapt the intervention. Independent of a single or multiple intervention tailoring, further data collection and analysis should be carried out following the temporal logic of the framework. However, this holistic application of all stages has not yet been used in precision prevention in occupational health (25). It is also possible to conduct continuous *Data Generation* with a *Data Management Lifecycle* independently of the development and implementation of the intervention, for example, to determine changes in selected factors of the employee over time.

### 4.4 Further development of the integrative conceptual framework

In the last step, perspectives for the application and further development of the integrative conceptual framework will be presented. From a micro-perspective, possibilities in the various stages of the integrative conceptual framework are first focused on and thus within the working environment. The focus is then extended to a meso and macro perspective and it is shown how the integrative conceptual framework could be further developed beyond the workplace setting, for example, in connection with other settings (e.g., family, community) and from an ecological approach.

#### 4.4.1 Micro perspective—data generation

As the proposed integrative conceptual framework is very general and, therefore, abstract, it can be concretized in all stages, which can help further deepen individual aspects and test them in empirical studies. Such a focus is recommended within collecting the determinants in the person/employee and the environment/workplace determinants or, ideally, for the combination of these two areas. The specification and selection of factors can be based on the models presented in Section 3.2, mainly the *Workplace Health Models*. Furthermore, numerous publications on precision prevention (12, 25), as well as reviews or studies on health in the workplace (worksite health promotion), can also be used for concretization (136, 137). The aim should be to specify and select determinants of the person/employee to represent a holistic understanding of health according to the biopsychosocial model (46). Thus, psychosocial and behavioral determinants are included in addition to biological ones. Both the publications by Hall (44) and Hekler et al. (3) and the review by Mess et al. (25) show that research on precision prevention is currently still too one-sided and strongly influenced by medical/biological thinking when selecting determinants. For example, in the scoping review by Mess et al. (25), it became clear, among other things, that almost half of the articles on assessment (data generation) focused on the employee’s risks, while only 22 percent of the articles addressed the employee’s resources. It would also be conceivable to select determinants about specific health-related behaviors [e.g., physical activity, dietary behavior (138–141)] or specific health outcomes [e.g., mental health, musculoskeletal diseases (142–144)]. For example, Lecours et al. (145) analyzed the antecedents and consequences of prevention behavior (in general) in their concept analysis and presented the determinants relevant to behavior in particular. With a specific focus on health-related behaviors, using the *Intervention Design and Evaluation Models* presented in Section 3.2.2 for further concretization would also be helpful. Among other things, these models propose systematic procedures (e.g., including *Intervention Mapping* and the *PRECEDE-PROCEED Model*) that can be used to identify determinants in a structured manner.

Such a focus and specification would also be helpful and essential for the determinants in the environment/workplace domain. The models analyzed in Section 3.2, mainly the *Workplace Health Models* and *Stress-Coping-Well-Being Theories*, can be used as an initial orientation and basis for selecting such determinants [e.g., (129)]. For further specification, however, the specific and necessary determinants can be chosen depending on the objective, target group or the intended health outcomes (129, 143, 146–152).

#### 4.4.2 Micro perspective—data management lifecycle

In the *Data Management Lifecycle* stage (collection, processing, storage, analytics, interpretation, inference), the integrative conceptual framework initially only contains very general indications, which can be concretized and expanded in further steps. The necessity and further possibilities for this are supported on the one hand by the qualitative content analyses (Sections 3.1.5 and 4.1). On the other hand, there are also many other publications, both in general research and in precision prevention research, which deal specifically with various concepts and approaches in the area of data analytics, such as profiling [e.g., phenotyping (153, 154)], machine learning (155–158), [AI (159, 160), etc.] and provide numerous in-depth insights and methodological approaches. In addition to this fundamental further development of data analytics, it will also be important in the future that all data, which is usually still collected in different departments in companies and rarely merged (161), is brought together at a central point in the company and managed centrally under ethical, legal and social guidelines. This can be realized, for example, in a health portal, dashboard or health information system (42, 105, 162). Furthermore, qualitative health data (e.g., opinions, views, etc.) should be collected and used for profiling (103). Otherwise, precision prevention remains inaccurate, and both scientists and practitioners lack a crucial component for accurately promoting the health of individuals (1).

#### 4.4.3 Micro perspective—intervention (development, implementation adaptation)

The possibilities for developing, implementing and conducting interventions in the context of precision prevention in occupational health are diverse and far exceed the scope of this conceptual analysis. For this reason, only a few general and non-exhaustive approaches for deepening and expanding the integrative conceptual framework are outlined below. Firstly, specification and extension can be done using the models presented in Section 3.2, mainly the *Intervention Design and Evaluation Models* (e.g., *Intervention Mapping*, *RE-AIM Framework*; Section 3.2.2). These models offer additional aspects for developing and implementing interventions with different foci. Secondly, the insights gained in the qualitative content analysis (Sections 3.1 and 4.1) also show further deepening and expansion of the integrative conceptual framework, e.g., about the type of feedback (“provided aspects”) given to the employees. Thirdly, other publications on developing, implementing and realizing precise interventions can be consulted. When developing interventions, for example, it is essential to select the most suitable one for the research question from many designs (163) and associated types of interventions (3, 164). In the future, the focus of the interventions could be on JITAIs, *N*-of-1 trials or MRTs, as these achieve the highest degree of precision. They can also usually be adapted several times during implementation/realization. Such intervention designs can provide valuable findings for optimizing and adapting intervention components for individuals in their respective contexts (3). More precise specifications can also be made for implementing interventions, such as identifying factors that can improve the realization in real-world settings (21, 165). Publications that present frameworks and models for implementing interventions in workplace settings (166) or the healthcare system (167) are also helpful for further focus.

#### 4.4.4 Meso and macro perspective

Taking into account the *Health-In-All Policies* of the WHO (168, 169) or, for example, the *Social Ecological Model of Health* (95, 170), it is essential to consider aspects outside a setting in the sense of holistic prevention or a multisectoral approach (169, 171). A future expansion of the integrative conceptual framework for precision prevention in occupational health can, therefore, take place in two further perspectives, among others: Firstly, the integrative conceptual framework can be linked with other relevant settings of the employee, for example, with the family or the community (meso perspective). Several publications already link different settings in this way, for example, as part of health promotion programs, particularly in the workplace and the family setting (172–175). These studies show, among other things, that it is essential for employees to involve their family members in occupational health promotion programs, as they would otherwise prefer to spend their free time with their family in the interests of work-family balance (175). In addition, these studies have shown that, for example, programs to promote healthy eating habits in the company can also positively affect fruit and vegetable consumption in the family (172, 173). In this respect, there appears to be a connection between health behavior at work and in the family environment, or rather a reciprocal influence.

Secondly, the proposed integrative conceptual framework can also take up ecological approaches [e.g., (95)] and thus be linked and extended to other overarching environmental conditions, for example, the general socioeconomic, cultural and ecological conditions (macro perspective). Such ecological approaches emphasize the importance of dynamic interactions between the individual, the workplace and the community (176). Precision prevention in occupational health is fundamentally firmly focused on the individual, and therefore, interventions are primarily aimed at the health-related behavior of the employee. However, in the future, the focus should shift more strongly to the occupational environment, such as working conditions (145). Interventions in the sense of multi-component programs should also be aimed at changing these environmental conditions (177–179). In this respect, it is essential to further develop the proposed integrative conceptual framework in the future about human–environment interactions and, for example, to include aspects of exposome research (6, 46, 180) or the integration into community settings (181). To this end, it would be important to pay even more attention to behavioral and social science as well as exposome research in the future, as recommended by Hall (44), Hekler et al. (3) and Delpierre et al. (46).

### 4.5 Limitations

This conceptual analysis is the first publication to systematically summarize the definitions and frameworks used in precision prevention in occupational health and to develop and propose a unified understanding and integrative conceptual framework for precision prevention in occupational health. Nevertheless, the analysis also has some methodological and conceptual limitations. Concerning the methodological approach, it should be critically noted, for example, that although we used a comprehensive search term and numerous databases for the search, we cannot exclude the possibility that further relevant studies could not be identified. For example, we included only original articles in the analyses and did not consider various other types of publications (books, book chapters, dissertations, grey literature, etc.). In addition, we have also focused only on English-language publications. A further limitation is that, although the articles were screened in pairs, the data extraction was carried out by one researcher each, which can lead to inaccuracies in the extracted data. Even though we proceeded according to standard guidelines in the qualitative content analysis (49) and, for example, categories (coding) were formed and compared by two independent researchers, it is still possible that subjective bias may have occurred.

One of the conceptual limitations is that the development of the integrative conceptual framework was not completely systematical and not all steps of the concept analysis research design were considered, as suggested by Walker et al. (182). Therefore, we may not have considered some crucial aspects, frameworks or models. Although conceptual review papers recommend such an explorative approach (47, 48, 50), it would be conceivable to carry out these steps systematically in the future and thus achieve greater generality and objectivity. Finally, when developing the integrative conceptual framework, findings from other disciplines of occupational health, e.g., occupational safety, could have been given greater consideration to present an even more holistic framework model.

### 4.6 Implications for research and practice

Researchers should critically examine, modify or expand the proposed unified understanding and the developed integrative conceptual framework of precision prevention in occupational health and thus contribute to its further development. It would also be desirable for researchers to derive more specific models based on the integrative conceptual framework to test these in empirical studies. The empirical findings could then, in turn, contribute to the further development of the integrative conceptual framework. Such empirical studies in companies could help researchers and practitioners critically discuss the practicability and applicability of the integrative conceptual framework together. This could fundamentally expand knowledge of precision prevention in occupational health and, thus, among those responsible for health (6). This could ultimately lead to an increase in mostly meager participation rates in workplace health promotion programs (183).

The integrative conceptual framework can improve professional knowledge and practice and thus help those involved in operational practice to further develop the occupational health and safety management for which they are responsible, both in content and structure. In terms of content, the proposed integrative conceptual framework can help practitioners, for example, to check whether their developed health assessment is complete and whether they have collected all the relevant (health) information from the employees and to develop it further if necessary. In terms of content, the framework can also help to integrate feedback loops or counseling, even more specifically, into occupational health and safety management, for example, to give employees feedback on their personal health and, at the same time, provide them with even more targeted advice for individual health promotion programs. Structurally, the integrative conceptual framework can support those involved in operational practice in bringing together the numerous employee data available at a central department and analyzing them systematically and continuously (e.g., in controlling). This could help reduce the challenges associated with using data from heterogeneous data sources and make data collection and analysis more effective (135). In addition, the practical application could result in relevant findings regarding implementation and feasibility, which other practitioners can, in turn, use as a guide to good or best practice (184). These practical experiences could, in turn, be taken up by the scientific community and considered when planning future studies.

A future challenge in precision prevention research and practice will also be to organize the handling of large amounts of data following ethical (91) and data security regulations (185). In addition to a big data approach, appropriate methods for a small data approach (the use of data by and for a specific *N*-of-1 unit, e.g., a single organization, unit or person, etc.) should also be further refined in the future and strategies for better integration of a small data approach in practice should be developed (186). From a methodological perspective, it would be advisable to further develop the unified understanding and integrative conceptual framework with additional methodological procedures, for example, through expert surveys or Delphi studies (187) or by using ontologies [frameworks that provide controlled vocabularies to help unify and connect scientific fields (188)].

## 5 Conclusion

This conceptual analysis is a first attempt to provide further insight and better orientation in precision prevention in occupational health, especially regarding a unified understanding and an integrative conceptual framework. The 154 identified studies show that this research field has developed rapidly, especially in recent years. However, the analyses also reveal that there is still a great deal of heterogeneity in the definitions or descriptions, understandings and models used to date. In this respect, the unified understanding and the integrative conceptual framework developed in this article should only be understood as a proposal or draft, as a starting point for further discussions and developments. Therefore, the future aim should be to critically examine and discuss the unified understanding and integrative conceptual framework of precision prevention in occupational health and develop it further in cooperation between science and practice. In addition to this critical discussion and further development, it would also be desirable for stakeholders in research and practice to differentiate and concretize the integrative conceptual framework for specific objectives to subsequently test it in empirical studies. For example, application to specific target groups (e.g., blue-collar workers, office workers, etc.) in different companies (sectors) or cultures could contribute to the continuous development of precision prevention in occupational health and thus attract even more attention in the future. This could ultimately bring significant health benefits to both employees and companies, and thus to our society, and promote efforts in prevention as a whole.

## Data Availability

The original contributions presented in the study are included in the article/Supplementary material, further inquiries can be directed to the corresponding author.

## Footnotes

^1^ http://rayyan.qcri.org
