# Supplementary material for: Precision prevention in occupational health: a conceptual analysis and development of a unified understanding and an integrative framework
Source: Front Public Health. 2024 Sep 18;12:1444521. doi: 10.3389/fpubh.2024.1444521 (PMC11445082; doi:10.3389/fpubh.2024.1444521)
Supplement: Supplementary file 1 [file Table_1.DOCX]

Supplementary Material

Precision prevention in occupational health: A conceptual analysis and development of a unified understanding and an integrative framework

Filip Mess^1^, Simon Blaschke^1^, Doris Gebhard^1^, Julian Friedrich^1^*

^1^ Technical University of Munich, TUM School of Medicine and Health, Department Health and Sport Sciences, Munich, Germany

*** Correspondence:** Julian Friedrich: julian.friedrich@tum.de

**S1 Table 1.** Search strings.

| **Web of Science**^TM^ |
| --- |
| (TI = (workplace or “work place” or worksite or “work site” or organisational or organizational or occupational* or worker or employee or corporate) AND TI = ((precision or personali* or individuali* or stratif* or Tailo* or target*) NEAR/0(health or intervention or program or prevention or “health promotion”) )) or (AB = (workplace or “work place” or worksite or “work site” or organisational or organizational or occupational* or worker or employee or corporate) AND AB = ((precision or personali* or individuali* or stratif* or tailo* or target*) NEAR/0(health or intervention or program or prevention or “health promotion”))) |
| **Scopus**^®^ |
| ( TITLE ( workplace OR "work place" OR worksite OR "work site" OR organisational OR organizational OR occupational* OR worker OR employee OR corporate ) AND ( TITLE ( precision OR personali* OR individuali* OR stratif* OR tailo* OR target* ) PRE/0 ( health OR intervention OR program OR prevention OR "health promotion" ) ) ) OR ( ABS ( workplace OR "work place" OR worksite OR "work site" OR organisational OR organizational OR occupational* OR worker OR employee OR corporate ) AND ABS ( ( precision OR personali* OR individuali* OR stratif* OR tailo* OR target* ) PRE/0 ( health OR intervention OR program OR prevention OR "health promotion" ))) |

| **Ovid MEDLINE**^®^ | |
| --- | --- |
| health | (((workplace or work place or worksite or work site or organisational or organizational or occupational* or worker or employee or corporate) and ((precision or personali* or individuali* or stratif* or tailo* or target*) adj health)).ti. or (workplace or work place or worksite or work site or organisational or organizational or occupational* or worker or employee or corporate).ab.) and ((precision or personali* or individuali* or stratif* or tailo* or target*) adj health).ab. |
| intervention | (((workplace or work place or worksite or work site or organisational or organizational or occupational* or worker or employee or corporate) and ((precision or personali* or individuali* or stratif* or Tailo* or target*) adj intervention)).ti. or (workplace or work place or worksite or work site or organisational or organizational or occupational* or worker or employee or corporate).ab.) and ((precision or personali* or individuali* or stratif* or tailo* or target*) adj intervention).ab. |
| program | (((workplace or work place or worksite or work site or organisational or organizational or occupational* or worker or employee or corporate) and ((precision or personali* or individuali* or stratif* or Tailo* or target*) adj program)).ti. or (workplace or work place or worksite or work site or organisational or organizational or occupational* or worker or employee or corporate).ab.) and ((precision or personali* or individuali* or stratif* or tailo* or target*) adj program).ab. |
| prevention | (((workplace or work place or worksite or work site or organisational or organizational or occupational* or worker or employee or corporate) and ((precision or personali* or individuali* or stratif* or Tailo* or target*) adj prevention)).ti. or (workplace or work place or worksite or work site or organisational or organizational or occupational* or worker or employee or corporate).ab.) and ((precision or personali* or individuali* or stratif* or tailo* or target*) adj prevention).ab. |
| health promotion | (((workplace or work place or worksite or work site or organisational or organizational or occupational* or worker or employee or corporate) and ((precision or personali* or individuali* or stratif* or Tailo* or target*) adj "health promotion")).ti. or (workplace or work place or worksite or work site or organisational or organizational or occupational* or worker or employee or corporate).ab.) and ((precision or personali* or individuali* or stratif* or tailo* or target*) adj "health promotion").ab. |

| **PubMed**^®^ | |
| --- | --- |
| precision | (workplace[Title/Abstract] or "work place" [Title/Abstract] or worksite [Title/Abstract] or "work site" [Title/Abstract] organisational [Title/Abstract] or organizational [Title/Abstract] or occupational [Title/Abstract] or worker [Title/Abstract] or employee [Title/Abstract] or corporate [Title/Abstract]) and ("precision health" [Title/Abstract] or "precision prevention" [Title/Abstract] or "precision intervention" [Title/Abstract] or "precision program" [Title/Abstract] or "precision health promotion" [Title/Abstract]) |
| personalized and personalised | (workplace[Title/Abstract] or "work place" [Title/Abstract] or worksite [Title/Abstract] or "work site" [Title/Abstract] organisational [Title/Abstract] or organizational [Title/Abstract] or occupational [Title/Abstract] or worker [Title/Abstract] or employee [Title/Abstract] or corporate [Title/Abstract]) and ("personalized health" [Title/Abstract] or "personalized prevention" [Title/Abstract] or "personalized intervention" [Title/Abstract] or "personalized program" [Title/Abstract] or "personalized health promotion" [Title/Abstract] or "personalised health" [Title/Abstract] or "personalised prevention" [Title/Abstract] or "personalised intervention" [Title/Abstract] or "personalised program" [Title/Abstract] or "personalised health promotion" [Title/Abstract]) |
| individualized and individualised | (workplace[Title/Abstract] or "work place" [Title/Abstract] or worksite [Title/Abstract] or "work site" [Title/Abstract] organisational [Title/Abstract] or organizational [Title/Abstract] or occupational [Title/Abstract] or worker [Title/Abstract] or employee [Title/Abstract] or corporate [Title/Abstract]) and ("individualized health" [Title/Abstract] or " individualized prevention" [Title/Abstract] or " individualized intervention" [Title/Abstract] or " individualized program" [Title/Abstract] or " individualized health promotion" [Title/Abstract] or "individualised health" [Title/Abstract] or " individualised prevention" [Title/Abstract] or " individualised intervention" [Title/Abstract] or " individualised program" [Title/Abstract] or " individualised health promotion" [Title/Abstract]) |
| stratified | (workplace [Title/Abstract] or "work place" [Title/Abstract] or worksite [Title/Abstract] or "work site" [Title/Abstract] organisational [Title/Abstract] or organizational [Title/Abstract] or occupational [Title/Abstract] or worker [Title/Abstract] or employee [Title/Abstract] or corporate [Title/Abstract]) and ("stratified health" [Title/Abstract] or " stratified prevention" [Title/Abstract] or " stratified intervention" [Title/Abstract] or " stratified program" [Title/Abstract] or " stratified health promotion" [Title/Abstract]) |
| tailored | (workplace[Title/Abstract] or "work place" [Title/Abstract] or worksite [Title/Abstract] or "work site" [Title/Abstract] organisational [Title/Abstract] or organizational [Title/Abstract] or occupational [Title/Abstract] or worker [Title/Abstract] or employee [Title/Abstract] or corporate [Title/Abstract]) and ("tailored health" [Title/Abstract] or " tailored prevention" [Title/Abstract] or " tailored intervention" [Title/Abstract] or " tailored program" [Title/Abstract] or " tailored health promotion" [Title/Abstract]) |
| targeted | (workplace[Title/Abstract] or "work place" [Title/Abstract] or worksite [Title/Abstract] or "work site" [Title/Abstract] organisational [Title/Abstract] or organizational [Title/Abstract] or occupational [Title/Abstract] or worker [Title/Abstract] or employee [Title/Abstract] or corporate [Title/Abstract]) and ("targeted health" [Title/Abstract] or " targeted prevention" [Title/Abstract] or " targeted intervention" [Title/Abstract] or " targeted program" [Title/Abstract] or " targeted health promotion" [Title/Abstract]) |

| **APA PsychInfo**^®^ **(via Scopus**^®^**)** | |
| --- | --- |
| personalized | AB ( workplace or work place or worksite or work site or organisational or organizational or occupational* or worker or employee or corporate ) AND AB ( “personalized health” or “personalized intervention” or “personalized program” or “personalized prevention” or “personalized health promotion” ) OR TI ( workplace or work place or worksite or work site or organisational or organizational or occupational* or worker or employee or corporate ) AND TI ( “personalized health” or “personalized intervention” or “personalized program” or “personalized prevention” or “personalized health promotion” ) |
| personalised | AB ( workplace or work place or worksite or work site or organisational or organizational or occupational* or worker or employee or corporate ) AND AB ( “personalised health” or “personalised intervention” or “personalised program” or “personalised prevention” or “personalised health promotion” ) OR TI ( workplace or work place or worksite or work site or organisational or organizational or occupational* or worker or employee or corporate ) AND TI ( “personalised health” or “personalised intervention” or “personalised program” or “personalised prevention” or “personalised health promotion” ) |
| precision | AB ( workplace or work place or worksite or work site or organisational or organizational or occupational* or worker or employee or corporate ) AND AB ( “precision health” or “precision intervention” or “precision program” or “precision prevention” or “precision health promotion” ) OR TI ( workplace or work place or worksite or work site or organisational or organizational or occupational* or worker or employee or corporate ) AND TI ( “precision health” or “precision intervention” or “precision program” or “precision prevention” or “precision health promotion” ) |
| targeted | AB ( workplace or work place or worksite or work site or organisational or organizational or occupational* or worker or employee or corporate ) AND AB ( “targeted health” or “targeted intervention” or “targeted program” or “targeted prevention” or “targeted health promotion” ) OR TI ( workplace or work place or worksite or work site or organisational or organizational or occupational* or worker or employee or corporate ) AND TI ( “targeted health” or “targeted intervention” or “targeted program” or “targeted prevention” or “targeted health promotion” ) |
| tailored | AB ( workplace or work place or worksite or work site or organisational or organizational or occupational* or worker or employee or corporate ) AND AB ( “tailored health” or “tailored intervention” or “tailored program” or “tailored prevention” or “tailored health promotion” ) OR TI ( workplace or work place or worksite or work site or organisational or organizational or occupational* or worker or employee or corporate ) AND TI ( “tailored health” or “tailored intervention” or “tailored program” or “tailored prevention” or “tailored health promotion” ) |
| stratified | AB ( workplace or work place or worksite or work site or organisational or organizational or occupational* or worker or employee or corporate ) AND AB ( “stratified health” or “stratified intervention” or “stratified program” or “stratified prevention” or “stratified health promotion” ) OR TI ( workplace or work place or worksite or work site or organisational or organizational or occupational* or worker or employee or corporate ) AND TI ( “stratified health” or “stratified intervention” or “stratified program” or “stratified prevention” or “stratified health promotion” ) |
| individualized | AB ( workplace or work place or worksite or work site or organisational or organizational or occupational* or worker or employee or corporate ) AND AB ( “individualized health” or “individualized intervention” or “individualized program” or “individualized prevention” or “individualized health promotion” ) OR TI ( workplace or work place or worksite or work site or organisational or organizational or occupational* or worker or employee or corporate ) AND TI ( “individualized health” or “individualized intervention” or “individualized program” or “individualized prevention” or “individualized health promotion” ) |
| individualised | AB ( workplace or work place or worksite or work site or organisational or organizational or occupational* or worker or employee or corporate ) AND AB ( “individualised health” or “individualised intervention” or “individualised program” or “individualised prevention” or “individualised health promotion” ) OR TI ( workplace or work place or worksite or work site or organisational or organizational or occupational* or worker or employee or corporate ) AND TI ( “individualised health” or “individualised intervention” or “individualised program” or “individualised prevention” or “individualised health promotion” ) |

| **Embase**^®^ **(via OVID)** | |
| --- | --- |
| health | ((workplace or work place or worksite or work site or organisational or organizational or occupational* or worker or employee or corporate) and ((precision or personali* or individuali* or stratif* or tailo* or target*) adj health)).ti. or ((workplace or work place or worksite or work site or organisational or organizational or occupational* or worker or employee or corporate) and ((precision or personali* or individuali* or stratif* or tailo* or target*) adj health)).ab. |
| intervention | ((workplace or work place or worksite or work site or organisational or organizational or occupational* or worker or employee or corporate) and ((precision or personali* or individuali* or stratif* or tailo* or target*) adj intervention)).ti. or ((workplace or work place or worksite or work site or organisational or organizational or occupational* or worker or employee or corporate) and ((precision or personali* or individuali* or stratif* or tailo* or target*) adj intervention)).ab. |
| program | ((workplace or work place or worksite or work site or organisational or organizational or occupational* or worker or employee or corporate) and ((precision or personali* or individuali* or stratif* or Tailo* or target*) adj program)).ti. or ((workplace or work place or worksite or work site or organisational or organizational or occupational* or worker or employee or corporate) and ((precision or personali* or individuali* or stratif* or tailo* or target*) adj program)).ab. |
| prevention | ((workplace or work place or worksite or work site or organisational or organizational or occupational* or worker or employee or corporate) and ((precision or personali* or individuali* or stratif* or Tailo* or target*) adj prevention)).ti. or ((workplace or work place or worksite or work site or organisational or organizational or occupational* or worker or employee or corporate) and ((precision or personali* or individuali* or stratif* or tailo* or target*) adj prevention)).ab. |
| health promotion | ((workplace or work place or worksite or work site or organisational or organizational or occupational* or worker or employee or corporate) and ((precision or personali* or individuali* or stratif* or Tailo* or target*) adj health promotion)).ti. or ((workplace or work place or worksite or work site or organisational or organizational or occupational* or worker or employee or corporate) and ((precision or personali* or individuali* or stratif* or tailo* or target*) adj health promotion)).ab. |

**S1. Table 2.** Eligibility criteria.

|  | **Inclusion** | **Exclusion** |
| --- | --- | --- |
| Population | - Human participants: workers, employees, trainees, etc., in the “working context” (workplace, worksite, organization, occupation, etc.) of any organization/company, etc. - Analysis of historical datasets (e.g., health records, epidemiological datasets) - Human samples (e.g., tissue samples, genetic material) | - Human participants outside the “working context” (e.g., community, school, health care settings: patients, military, etc.); children - Evaluation of new technologies that do not include human participants in the working context (cf. Ryan et al., 2021) - Human/sports performance outcomes (e.g., physical conditioning programs for healthy athletes) |
| Concept | - Studies that refer to the concept of precision prevention or its derivates (cf. search term) - Any study collecting health-related clinical, psychosocial, behavioral, environmental and work-related information (e.g. weight loss, disease prevalence/risk, healthy diet, physical activity, sedentariness, mental health) | - Non-health outcomes including economic outcomes (e.g., cost-effectiveness studies) - Animal studies |
| Context | - Any geographical location - Setting: workplace etc. (see. above) | - “non-occupational settings/other settings” (community, school, etc.) |
| Types of evidence | - Primary empirical research studies (e.g., RCTs, cohort studies, cross, sectional studies, and case reports) - Protocols for planned studies - Full-text articles available in electronic or hard copy - Full-text conference proceedings - Articles written in English - Peer-reviewed journal papers | - Reviews, meta-analyses  (e.g., systematic, narrative, scoping reviews) - Editorial articles  (e.g., position statements) - Protocols for reviews - Abstracts or posters - Articles: full-text not available in electronic or hard-copy - Articles (full text) not written in English - Dissertations, book chapters, books, etc. |
