# Supplementary material for: Precision prevention in occupational health: a conceptual analysis and development of a unified understanding and an integrative framework
Source: Front Public Health. 2024 Sep 18;12:1444521. doi: 10.3389/fpubh.2024.1444521 (PMC11445082; doi:10.3389/fpubh.2024.1444521)
Supplement: Supplementary file 2 [file Table_2.DOCX]

Supplementary Material

Precision prevention in occupational health: A conceptual analysis and development of a unified understanding and an integrative framework

Filip Mess^1^, Simon Blaschke^1^, Doris Gebhard^1^, Julian Friedrich^1^*

^1^ Technical University of Munich, TUM School of Medicine and Health, Department Health and Sport Sciences, Munich, Germany

*** Correspondence:** Julian Friedrich: julian.friedrich@tum.de

**S2.** Reference list of included articles.

1. Abbas SZ, Pollard TM, Wynn P, Learmonth A, Joyce K, Bambra C. The effectiveness of using the workplace to identify and address modifiable health risk factors in deprived populations. Occup Environ Med. 2015;72(9):664-9.

2. Adorjan K, Dong MS, Wratil PR, Schmacke NA, Weinberger T, Steffen J, et al. Development and Validation of a Simple Tool for Predicting Pandemic-Related Psychological Distress Among Health Care Workers. Journal of Technology in Behavioral Science. 2024.

3. Ammar S, Daud A, Ismail AF, Razali A. Efficacy of a Targeted Intervention Method to Improve the Use of Hearing Protection Devices among Agro-Industrial Workers in Malaysia. Applied Sciences. 2022;12(5).

4. Andersen LN, Juul-Kristensen B, Roessler KK, Herborg LG, Sorensen TL, Sogaard K. Efficacy of 'Tailored Physical Activity' in reducing sickness absence among health care workers: design of a randomised controlled trial. BMC Public Health. 2013;13(1):917.

5. Andersen LN, Juul-Kristensen B, Roessler KK, Herborg LG, Sorensen TL, Sogaard K. Efficacy of 'Tailored Physical Activity' on reducing sickness absence among health care workers: A 3-months randomised controlled trial. Man Ther. 2015;20(5):666-71.

6. Andersen LN, Juul-Kristensen B, Sorensen TL, Herborg LG, Roessler KK, Sogaard K. Efficacy of Tailored Physical Activity or Chronic Pain Self-Management Programme on return to work for sick-listed citizens: A 3-month randomised controlled trial. Scand J Public Health. 2015;43(7):694-703.

7. Andersson M, Nordin A, Fredholm A, Engstrom A. The four domains of the person-centred practice framework from the perspective of critical care nurses in intensive care units during a pandemic. Intensive Crit Care Nurs. 2023;78:103449.

8. Asfar T, Arheart KL, McClure LA, Ruano-Herreria EC, Dietz NA, Ward KD, et al. Implementing a Novel Workplace Smoking Cessation Intervention Targeting Hispanic/Latino Construction Workers: A Pilot Cluster Randomized Trial. Health Educ Behav. 2021;48(6):795-804.

9. Asfar T, Caban-Martinez AJ, McClure LA, Ruano-Herreria EC, Sierra D, Gilford Clark G, Jr., et al. A cluster randomized pilot trial of a tailored worksite smoking cessation intervention targeting Hispanic/Latino construction workers: Intervention development and research design. Contemp Clin Trials. 2018;67:47-55.

10. Baumann H, Heuel L, Bischoff LL, Wollesen B. Efficacy of Individualized Sensory-Based mHealth Interventions to Improve Distress Coping in Healthcare Professionals: A Multi-Arm Parallel-Group Randomized Controlled Trial. Sensors (Basel). 2023;23(4):2322.

11. Baumann H, Heuel L, Bischoff LL, Wollesen B. mHealth interventions to reduce stress in healthcare workers (fitcor): study protocol for a randomized controlled trial. Trials. 2023;24(1):163.

12. Beer-Borst S, Hayoz S, Eisenblatter J, Jent S, Siegenthaler S, Strazzullo P, et al. RE-AIM evaluation of a one-year trial of a combined educational and environmental workplace intervention to lower salt intake in Switzerland. Prev Med Rep. 2019;16:100982.

13. Béjar LM, Mesa-Rodriguez P, Garcia-Perea MD. Short-Term Effect of a Health Promotion Intervention Based on the Electronic 12-Hour Dietary Recall (e-12HR) Smartphone App on Adherence to the Mediterranean Diet Among Spanish Primary Care Professionals: Randomized Controlled Clinical Trial. JMIR Mhealth Uhealth. 2024;12:e49302.

14. Berninger NM, Plasqui G, Crutzen R, Ruiter RAC, Kok G, Ten Hoor GA. The Effects of UPcomplish on Office Workers' Sedentary Behaviour, Quality of Life and Psychosocial Determinants: A Stepped-Wedge Design. Int J Behav Med. 2022;29(6):728-42.

15. Berninger NM, Ten Hoor GA, Plasqui G, Kok G, Peters GY, Ruiter RAC. Sedentary Work in Desk-Dominated Environments: A Data-Driven Intervention Using Intervention Mapping. JMIR Form Res. 2020;4(7):e14951.

16. Bidargaddi N, Almirall D, Murphy S, Nahum-Shani I, Kovalcik M, Pituch T, et al. To Prompt or Not to Prompt? A Microrandomized Trial of Time-Varying Push Notifications to Increase Proximal Engagement With a Mobile Health App. JMIR Mhealth Uhealth. 2018;6(11):e10123.

17. Blake H, Bennett E, E. Batt M. Evaluation of occupational health checks for hospital employees. International Journal of Workplace Health Management. 2014;7(4):247-66.

18. Blake H, Hussain B, Hand J, Rowlands D, Juma A, Evans C. Employee perceptions of a workplace HIV testing intervention. International Journal of Workplace Health Management. 2018;11(5):333-48.

19. Bonn S, Licitra G, Bellocco R, Trolle Lagerros Y. Clinical Outcomes Among Working Adults Using the Health Integrator Smartphone App: Analyses of Prespecified Secondary Outcomes in a Randomized Controlled Trial. J Med Internet Res. 2022;24(3):e24725.

20. Bonn SE, Lof M, Ostenson CG, Trolle Lagerros Y. App-technology to improve lifestyle behaviors among working adults - the Health Integrator study, a randomized controlled trial. BMC Public Health. 2019;19(1):273.

21. Broneder E, Wagner F, Weiß C, Fritz J, Sili M, Arendse M. mHealthINX – A Virtual Reality-Based Occupational Stress Management Solution for Older Employees. 2021. p. 249-56.

22. Brossoit RM, Hammer LB, Crain TL, Leslie JJ, Bodner TE, Brockwood KJ. The effects of a Total Worker Health intervention on workplace safety: Mediating effects of sleep and supervisor support for sleep. J Occup Health Psychol. 2023;28(4):263-76.

23. Caperchione CM, Sharp P, Bottorff JL, Stolp S, Oliffe JL, Johnson ST, et al. The POWERPLAY workplace physical activity and nutrition intervention for men: Study protocol and baseline characteristics. Contemp Clin Trials. 2015;44:42-7.

24. Caperchione CM, Stolp S, Bottorff JL, Oliffe JL, Johnson ST, Seaton C, et al. Changes in Men's Physical Activity and Healthy Eating Knowledge and Behavior as a Result of Program Exposure: Findings From the Workplace POWERPLAY Program. J Phys Act Health. 2016;13(12):1364-71.

25. Castel-Feced S, Malo S, Aguilar-Palacio I, Feja-Solana C, Casasnovas JA, Maldonado L, et al. Influence of cardiovascular risk factors and treatment exposure on cardiovascular event incidence: Assessment using machine learning algorithms. PLoS One. 2023;18(11):e0293759.

26. Chadyiwa M, Kagura J, Stewart A. Investigating Machine Learning Applications in the Prediction of Occupational Injuries in South African National Parks. Machine Learning and Knowledge Extraction. 2022;4(3):768-78.

27. Charzynska E, Habibi Soola A, Mozaffari N, Mirzaei A. Patterns of work-related stress and their predictors among emergency department nurses and emergency medical services staff in a time of crisis: a latent profile analysis. BMC Nurs. 2023;22(1):98.

28. Choi SH, Waltje AH, Ronis DL, Noonan D, Hong O, Richardson CR, et al. Web-enhanced tobacco tactics with telephone support versus 1-800-QUIT-NOW telephone line intervention for operating engineers: randomized controlled trial. J Med Internet Res. 2014;16(11):e255.

29. Christensen JR, Faber A, Ekner D, Overgaard K, Holtermann A, Sogaard K. Diet, physical exercise and cognitive behavioral training as a combined workplace based intervention to reduce body weight and increase physical capacity in health care workers - a randomized controlled trial. BMC Public Health. 2011;11(1):671.

30. Christensen JR, Overgaard K, Carneiro IG, Holtermann A, Sogaard K. Weight loss among female health care workers--a 1-year workplace based randomized controlled trial in the FINALE-health study. BMC Public Health. 2012;12(1):625.

31. Damen I, Van Den Heuvel R, Brankaert R, Vos S, editors. Advancing Digital Behavior Change Interventions by Exploring a Calendar-Based Suggestion System2021 2021.

32. Das BM, Petruzzello SJ. The use of active living every day to improve mass transit district employees' physical activity affect and enjoyment. International Journal of Health Promotion and Education. 2014;53(3):147-55.

33. Dashti HS, Alimenti K, Levy DE, Hivert MF, McCurley JL, Saxena R, et al. Chronotype Polygenic Score and the Timing and Quality of Workplace Cafeteria Purchases: Secondary Analysis of the ChooseWell 365 Randomized Controlled Trial. Curr Dev Nutr. 2023;7(3):100048.

34. De Cocker K, Cardon G, Bennie JA, Kolbe-Alexander T, Meester F, Vandelanotte C. From Evidence-Based Research to Practice-Based Evidence: Disseminating a Web-Based Computer-Tailored Workplace Sitting Intervention through a Health Promotion Organisation. Int J Environ Res Public Health. 2018;15(5):1049.

35. De Cocker K, Cardon G, Vergeer I, Radtke T, Vandelanotte C. Who Uses Action Planning in a Web-Based Computer-Tailored Intervention to Reduce Workplace Sitting and What do Action Plans Look Like? Analyses of the Start to stand Intervention among Flemish Employees. Appl Psychol Health Well Being. 2019;11(3):543-61.

36. De Cocker K, De Bourdeaudhuij I, Cardon G, Vandelanotte C. Theory-driven, web-based, computer-tailored advice to reduce and interrupt sitting at work: development, feasibility and acceptability testing among employees. BMC Public Health. 2015;15(1):959.

37. De Cocker K, De Bourdeaudhuij I, Cardon G, Vandelanotte C. The Effectiveness of a Web-Based Computer-Tailored Intervention on Workplace Sitting: A Randomized Controlled Trial. J Med Internet Res. 2016;18(5):e96.

38. De Cocker K, De Bourdeaudhuij I, Cardon G, Vandelanotte C. What are the working mechanisms of a web-based workplace sitting intervention targeting psychosocial factors and action planning? BMC Public Health. 2017;17(1):382.

39. Dias L, Eertmans A, Van den Brande I, Handaja Y, Taeymans S, Vansteenwegen D. Using an Interactive Self-Assessment Tool to Strengthen Your Employee Assistance Service. Journal of Workplace Behavioral Health. 2015;30(1-2):46-65.

40. Doda D, Rothmore P, Pisaniello D, Briggs N, Stewart S, Mahmood M, et al. Relative benefit of a stage of change approach for the prevention of musculoskeletal pain and discomfort: a cluster randomised trial. Occup Environ Med. 2015;72(11):784-91.

41. Duan Y, Shang B, Liang W, Yang M, Brehm W. Psychosocial profiles of physical activity fluctuation in office employees: A latent profile analysis. PLoS One. 2020;15(1):e0227182.

42. Dzubur E, Yu J, Hoffman J, Painter S, James R, Shah B. The Effect of a Digital Mental Health Program on Anxiety and Depression Symptoms: Retrospective Analysis of Clinical Severity. JMIR Form Res. 2023;7:e36596.

43. Elvitigala DS, Scholl PM, Suriyaarachchi H, Dissanayake V, Nanayakkara S, editors. StressShoe: A DIY Toolkit for just-in-time Personalised Stress Interventions for Office Workers Performing Sedentary Tasks2021 2021.

44. Fetherman DL, Cebrick-Grossman J. Use of the PRECEDE-PROCEED Model to Pilot an Occupational Physical Activity Intervention: Tailored Through a Community Partnership. Workplace Health Saf. 2023;71(8):367-74.

45. Filon LF, Maculan P, Crivellaro MA, Mauro M. Effectiveness of a Skin Care Program With a Cream Containing Ceramide C and a Personalized Training for Secondary Prevention of Hand Contact Dermatitis. Dermatitis. 2023;34(2):127-34.

46. Friedrich J, Munch AK, Thiel A, Voelter-Mahlknecht S, Sudeck G. Occupational resource profiles for an addressee orientation in occupational health management: a segmentation analysis. Front Psychol. 2023;14:1200798.

47. Fu H, Xia Z, Tan Y, Guo X. Influence of Cues on the Safety Hazard Recognition of Construction Workers during Safety Training: Evidence from an Eye-Tracking Experiment. Journal of Civil Engineering Education. 2024;150(1).

48. Gans KM, Risica PM, Dulin-Keita A, Mello J, Dawood M, Strolla LO, et al. Innovative video tailoring for dietary change: final results of the Good for you! cluster randomized trial. Int J Behav Nutr Phys Act. 2015;12:130.

49. Gearhart CA, McCarthy CJ, Lambert RG. Teachers' psychological stress and wellbeing during a pandemic: Exploring latent profiles. Sch Psychol. 2023.

50. Gellert GA, Montgomery S, Gellert TE, Gillett J, Kerekes A, Hole J. Employee Wellness in a Changing Climate: Environmental Heat Stress Driving Need for Targeted Health Promotion and Risk Reduction. Journal of Ecophysiology and Occupational Health. 2022;22(2):60-6.

51. Gram B, Holtermann A, Sogaard K, Sjogaard G. Effect of individualized worksite exercise training on aerobic capacity and muscle strength among construction workers--a randomized controlled intervention study. Scand J Work Environ Health. 2012;38(5):467-75.

52. Green S, Markaki A, Baird J, Murray P, Edwards R. Addressing Healthcare Professional Burnout: A Quality Improvement Intervention. Worldviews Evid Based Nurs. 2020;17(3):213-20.

53. Grigorescu S, Cazan AM, Rogozea L, Grigorescu DO. Original targeted therapy for the management of the burnout syndrome in nurses: an innovative approach and a new opportunity in the context of predictive, preventive and personalized medicine. EPMA J. 2020;11(2):161-76.

54. Grigorescu S, Cazan AM, Grigorescu OD, Rogozea LM. The role of the personality traits and work characteristics in the prediction of the burnout syndrome among nurses-a new approach within predictive, preventive, and personalized medicine concept. EPMA J. 2018;9(4):355-65.

55. Gryshchuk L, Campbell MA, Brunelle C, Doyle JN, Nero JW. Profiles of Vulnerability to Alcohol Use and Mental Health Concerns in First Responders. Journal of Police and Criminal Psychology. 2022;37(4):952-61.

56. Hao R, Zuo J, Jin H, Wang Y, Zhang L, Zhu Y, et al. Anxiety-associated factors among employees with different personality profiles: a cross-sectional study in China. Front Psychol. 2023;14:1043339.

57. Haslam C, Kazi A, Duncan M, Clemes S, Twumasi R. Walking Works Wonders: a tailored workplace intervention evaluated over 24 months. Ergonomics. 2018;62(1):31-41.

58. Haslam C, Kazi A, Duncan M, Twumasi R, Clemes S. Walking Works Wonders: A Workplace Health Intervention Evaluated Over 24 Months. 2019. p. 1571-8.

59. Hees HL, Koeter MW, de Vries G, Ooteman W, Schene AH. Effectiveness of adjuvant occupational therapy in employees with depression: design of a randomized controlled trial. BMC Public Health. 2010;10:558.

60. Hees HL, de Vries G, Koeter MW, Schene AH. Adjuvant occupational therapy improves long-term depression recovery and return-to-work in good health in sick-listed employees with major depression: results of a randomised controlled trial. Occup Environ Med. 2013;70(4):252-60.

61. Hnizdo E, Berry A, Hakobyan A, Beeckman-Wagner LA, Catlett L. Worksite wellness program for respiratory disease prevention in heavy-construction workers. J Occup Environ Med. 2011;53(3):274-81.

62. Holtermann A, Jorgensen MB, Gram B, Christensen JR, Faber A, Overgaard K, et al. Worksite interventions for preventing physical deterioration among employees in job-groups with high physical work demands: background, design and conceptual model of FINALE. BMC Public Health. 2010;10:120.

63. Hong O, Eakin BL, Chin DL, Feld J, Vogel S. An Internet-based tailored hearing protection intervention for firefighters: development process and users' feedback. Health Promot Pract. 2013;14(4):572-9.

64. Huang JJ, Lin HS, Yen M, Kan WM, Lee BO, Chen CH. Effects of a workplace multiple cardiovascular disease risks reduction program. Asian Nurs Res (Korean Soc Nurs Sci). 2013;7(2):74-82.

65. Hutchinson J, Headley S, Matthews T, Spicer G, Dempsey K, Wooley S, et al. Changes in Sitting Time and Sitting Fragmentation after a Workplace Sedentary Behaviour Intervention. Int J Environ Res Public Health. 2018;15(6).

66. Inchusri T, Surangsrirat D, Kwanmuang P, Poomivanichakij P, Apiwatgaroon P, Ongprakobkul S, et al. Association of Generation and Group Size With the Usage of a Mobile Health App in Thailand: Secondary Analysis of the ThaiSook Cohort Study. J Med Internet Res. 2023;25:e45374.

67. Jackson C, Smith S, Aghasaryan A, Andreasyan D, Aregay AK, Habersaat KB, et al. Barriers and drivers of positive COVID-19 vaccination behaviours among healthcare workers in Europe and Central Asia: a qualitative cross-country synthesis. Humanities and Social Sciences Communications. 2023;10(1).

68. Jay K, Brandt M, Sundstrup E, Schraefel M, Jakobsen MD, Sjogaard G, et al. Effect of individually tailored biopsychosocial workplace interventions on chronic musculoskeletal pain, stress and work ability among laboratory technicians: randomized controlled trial protocol. BMC Musculoskelet Disord. 2014;15:444.

69. Jay K, Brandt M, Hansen K, Sundstrup E, Jakobsen MD, schraefel m, et al. Effect of Individually Tailored Biopsychosocial Workplace Interventions on Chronic Musculoskeletal Pain and Stress Among Laboratory Technicians: Randomized Controlled Trial. Pain physician. 2015;18(5):459-71.

70. Jeelani I, Albert A, Han K, Azevedo R. Are Visual Search Patterns Predictive of Hazard Recognition Performance? Empirical Investigation Using Eye-Tracking Technology. Journal of Construction Engineering and Management. 2019;145(1).

71. Jensen JD, King AJ, Carcioppolo N, Krakow M, Samadder NJ, Morgan S. Comparing tailored and narrative worksite interventions at increasing colonoscopy adherence in adults 50-75: a randomized controlled trial. Soc Sci Med. 2014;104:31-40.

72. Jenull BB, Wiedermann W. The Different Facets of Work Stress: A Latent Profile Analysis of Nurses' Work Demands. J Appl Gerontol. 2015;34(7):823-43.

73. Jones-Bitton A, Gillis D, Peterson M, McKee H. Latent burnout profiles of veterinarians in Canada: Findings from a cross-sectional study. Vet Rec. 2023;192(2):e2281.

74. Kapanen AI, Conklin AI, Gobis B, Leung L, Yuen J, Zed PJ. Pharmacist-led cardiovascular risk prevention in Western Canada: a qualitative study. Int J Pharm Pract. 2021;29(1):45-54.

75. Kim JY, Oh S, Steinhubl S, Kim S, Bae WK, Han JS, et al. Effectiveness of 6 months of tailored text message reminders for obese male participants in a worksite weight loss program: randomized controlled trial. JMIR Mhealth Uhealth. 2015;3(1):e14.

76. Kocielnik R, Sidorova N. Personalized Stress Management: Enabling Stress Monitoring with LifelogExplorer. KI - Künstliche Intelligenz. 2015;29(2):115-22.

77. Kouwenhoven-Pasmooij TA, Robroek SJW, Kraaijenhagen RA, Helmhout PH, Nieboer D, Burdorf A, et al. Effectiveness of the blended-care lifestyle intervention 'PerfectFit': a cluster randomised trial in employees at risk for cardiovascular diseases. BMC Public Health. 2018;18(1):766.

78. Kraft D, Schmidt A, Büttner L, Oschinsky FM, Lambusch F, Van Laerhoven K, et al., editors. CareCam: Towards user-tailored Interventions at the Workplace using a Webcam2022 2022.

79. Kwasnicka D, Vandelanotte C, Rebar A, Gardner B, Short C, Duncan M, et al. Comparing motivational, self-regulatory and habitual processes in a computer-tailored physical activity intervention in hospital employees - protocol for the PATHS randomised controlled trial. BMC Public Health. 2017;17(1):518.

80. Kwasnicka D, Rebar A, elanotte C, Gardner B, Short C, Hagger M, et al. Physical activity tailored intervention in hospital (PATHS): A Randomised controlled trial on online tailored program. Annals of Behavioral Medicine. 2020;53(1):166.

81. Lamb JJ, Stone M, D'Adamo CR, Volkov A, Metti D, Aronica L, et al. Personalized Lifestyle Intervention and Functional Evaluation Health Outcomes SurvEy: Presentation of the LIFEHOUSE Study Using N-of-One Tent-Umbrella-Bucket Design. J Pers Med. 2022;12(1):115.

82. Langille JL, Berry TR, Reade IL, Witcher C, Loitz CC, Rodgers WM. Strength of messaging in changing attitudes in a workplace wellness program. Health Promot Pract. 2011;12(2):303-11.

83. Lawanot W, Inoue M, Yokemura T, Mongkolnam P, Nukoolkit C, editors. Daily Stress and Mood Recognition System Using Deep Learning and Fuzzy Clustering for Promoting Better Well-Being2019 2019.

84. Leduc C, Giga SI, Fletcher IJ, Young M, Dorman SC. Participatory Development Process of Two Human Dimension Intervention Programs to Foster Physical Fitness and Psychological Health and Well-Being in Wildland Firefighting. Int J Environ Res Public Health. 2021;18(13).

85. Leduc C, Giga SI, Fletcher IJ, Young M, Dorman SC. Effectiveness of fitness training and psychosocial education intervention programs in wildland firefighting: a cluster randomised control trial. Int J Wildland Fire. 2022;31(8):799-815.

86. Lenz TL, Monaghan MS. Implementing lifestyle medicine with medication therapy management services to improve patient-centered health care. J Am Pharm Assoc (2003). 2011;51(2):184-8.

87. Leung CLK, Li KK, Wei VWI, Tang A, Wong SYS, Lee SS, et al. Profiling vaccine believers and skeptics in nurses: A latent profile analysis. Int J Nurs Stud. 2022;126:104142.

88. Levy DE, Gelsomin ED, Rimm EB, Pachucki M, Sanford J, Anderson E, et al. Design of ChooseWell 365: Randomized controlled trial of an automated, personalized worksite intervention to promote healthy food choices and prevent weight gain. Contemp Clin Trials. 2018;75:78-86.

89. Li J, Yu H, He S, Xue M, Tian D, Zhou J, et al. The association between awareness and behavior concerning the need for protection when using pesticide sprays and neurologic symptoms: A latent class cluster analysis. Medicine (Baltimore). 2019;98(30):e16588.

90. Li Y, Wu J, Liu X, Zhang J, Zhong X, He L. Latent profile analysis and influence factors study of presenteeism among ICU nurses in China. Front Psychol. 2023;14:1259333.

91. Lieslehto J, Rantanen N, Oksanen LAH, Oksanen SA, Kivimaki A, Paju S, et al. A machine learning approach to predict resilience and sickness absence in the healthcare workforce during the COVID-19 pandemic. Sci Rep. 2022;12(1):8055.

92. Loeppke R, Edington D, Bender J, Reynolds A. The association of technology in a workplace wellness program with health risk factor reduction. J Occup Environ Med. 2013;55(3):259-64.

93. Marzocchi I, Ghezzi V, Di Tecco C, Ronchetti M, Ciampa V, Olivo I, et al. Demand-Resource Profiles and Job Satisfaction in the Healthcare Sector: A Person-Centered Examination Using Bayesian Informative Hypothesis Testing. Int J Environ Res Public Health. 2023;20(2):967.

94. Mauriello LM, Gokbayrak NS, Van Marter DF, Paiva AL, Prochaska JM. An Internet-Based Computer-Tailored Intervention to Promote Responsible Drinking: Findings from a Pilot Test with Employed Adults. Alcohol Treat Q. 2011;30(1):91-108.

95. McCall HC, Beahm JD, Fournier AK, Burnett JL, Carleton RN, Hadjistavropoulos HD. Stakeholder perspectives on internet-delivered cognitive behavioural therapy for public safety personnel: A qualitative analysis. Canadian Journal of Behavioural Science / Revue canadienne des sciences du comportement. 2021;53(3):232-42.

96. McGuckin T, Sealey R, Barnett F, Walla P. The use and evaluation of a theory-informed, multi-component intervention to reduce sedentary behaviour in the workplace. Cogent Psychology. 2017;4(1).

97. McHugh J, Suggs LS. Online tailored weight management in the worksite: does it make a difference in biennial health risk assessment data? J Health Commun. 2012;17(3):278-93.

98. McHugh SM, Riordan F, Kerins C, Curran G, Lewis CC, Presseau J, et al. Understanding tailoring to support the implementation of evidence-based interventions in healthcare: The CUSTOMISE research programme protocol. HRB Open Res. 2023;6:7.

99. Mistretta EG, Davis MC, Temkit M, Lorenz C, Darby B, Stonnington CM. Resilience Training for Work-Related Stress Among Health Care Workers: Results of a Randomized Clinical Trial Comparing In-Person and Smartphone-Delivered Interventions. J Occup Environ Med. 2018;60(6):559-68.

100. Monnaatsie M, Biddle SJH, Kolbe-Alexander T. The Feasibility of a Text-Messaging Intervention Promoting Physical Activity in Shift Workers: A Process Evaluation. Int J Environ Res Public Health. 2023;20(4):3260.

101. Niks I, de Jonge J, Gevers J, Houtman I. Work Stress Interventions in Hospital Care: Effectiveness of the DISCovery Method. Int J Environ Res Public Health. 2018;15(2).

102. Nylen EC, Lindfors P, Ishall L, Goransson S, Aronsson G, Kylin C, et al. A pilot-study of a worksite based participatory intervention program: Its acceptability and short-term effects on work climate and attitudes in human service employees. Work. 2017;56(4):625-36.

103. Otto AK, Wollesen B. Multicomponent exercises to prevent and reduce back pain in elderly care nurses: a randomized controlled trial. BMC Sports Sci Med Rehabil. 2022;14(1):114.

104. Ouellette RR, Frazier SL, Shernoff ES, Cappella E, Mehta TG, Marinez-Lora A, et al. Teacher Job Stress and Satisfaction in Urban Schools: Disentangling Individual-, Classroom-, and Organizational-Level Influences. Behav Ther. 2018;49(4):494-508.

105. Pandey A, Mishra RM, Sahu D, Benara SK, Sengupta U, Paranjape RS, et al. Heading towards the Safer Highways: an assessment of the Avahan prevention programme among long distance truck drivers in India. BMC Public Health. 2011;11 Suppl 6(Suppl 6):S15.

106. Patterson PD, Moore CG, Guyette FX, Doman JM, Sequeira D, Werman HA, et al. Fatigue mitigation with SleepTrackTXT2 in air medical emergency care systems: study protocol for a randomized controlled trial. Trials. 2017;18(1):254.

107. Patterson PD, Moore CG, Guyette FX, Doman JM, Weaver MD, Sequeira DJ, et al. Real-Time Fatigue Mitigation with Air-Medical Personnel: The SleepTrackTXT2 Randomized Trial. Prehosp Emerg Care. 2019;23(4):465-78.

108. Patterson PD, Moore CG, Weaver MD, Buysse DJ, Suffoletto BP, Callaway CW, et al. Mobile phone text messaging intervention to improve alertness and reduce sleepiness and fatigue during shiftwork among emergency medicine clinicians: study protocol for the SleepTrackTXT pilot randomized controlled trial. Trials. 2014;15(1):244.

109. Pesola AJ, Laukkanen A, Haakana P, Havu M, Saakslahti A, Sipila S, et al. Muscle inactivity and activity patterns after sedentary time--targeted randomized controlled trial. Med Sci Sports Exerc. 2014;46(11):2122-31.

110. Pidd K, Roche A, Fischer J. A recipe for good mental health: A pilot randomised controlled trial of a psychological wellbeing and substance use intervention targeting young chefs. Drugs: Education, Prevention and Policy. 2015;22(4):352-61.

111. Portoghese I, Siddi M, Chessa L, Costanzo G, Garcia-Larsen V, Perra A, et al. COVID-19 Vaccine Hesitancy among Italian Healthcare Workers: Latent Profiles and Their Relationships to Predictors and Outcome. Vaccines (Basel). 2023;11(2).

112. Portugal LCL, Gama CMF, Goncalves RM, Mendlowicz MV, Erthal FS, Mocaiber I, et al. Vulnerability and Protective Factors for PTSD and Depression Symptoms Among Healthcare Workers During COVID-19: A Machine Learning Approach. Front Psychiatry. 2021;12:752870.

113. Ratzon NZ, Bar-Niv NA, Froom P. The effect of a structured personalized ergonomic intervention program for hospital nurses with reported musculoskeletal pain: An assigned randomized control trial. Work. 2016;54(2):367-77.

114. Robles-Granda P, Lin S, Wu X, Martinez GJ, Mattingly SM, Moskal E, et al. Jointly Predicting Job Performance, Personality, Cognitive Ability, Affect, and Well-Being. IEEE Computational Intelligence Magazine. 2021;16(2):46-61.

115. Rollo S, Prapavessis H. A combined health action process approach and mHealth intervention to reduce workplace sitting time in office-working adults: a secondary analysis examining health-related quality of life and work performance outcomes. Psychol Health. 2021;36(10):1200-16.

116. Salinger MR, Levy DE, McCurley JL, Gelsomin ED, Rimm EB, Thorndike AN. Employees' Baseline Food Choices and the Effect of a Workplace Intervention to Promote Healthy Eating: Secondary Analysis of the ChooseWell 365 Randomized Controlled Trial. J Acad Nutr Diet. 2023;123(11):1586-95 e4.

117. Salmela J, Lahti J, Kanerva N, Rahkonen O, Kouvonen A, Lallukka T. Latent classes of unhealthy behaviours and their associations with subsequent sickness absence: a prospective register-linkage study among Finnish young and early midlife employees. BMJ Open. 2023;13(5):e070883.

118. Sanders MR, Stallman HM, McHale M. Workplace Triple P: A controlled evaluation of a parenting intervention for working parents. J Fam Psychol. 2011;25(4):581-90.

119. Schelleman-Offermans K, Ruiter RAC, Massar K. The Future Positive micro-intervention protocol: A program aiming to increase a healthy life-style among employees with a low socio-economic position. Front Public Health. 2022;10:832447.

120. Seaton CL, Bottorff JL, Oliffe JL, Jones-Bricker M, Caperchione CM, Johnson ST, et al. Acceptability of the POWERPLAY Program: A Workplace Health Promotion Intervention for Men. Am J Mens Health. 2017;11(6):1809-22.

121. Soliman GA, Kim J, Lee JM, High R, Hortman S, Kim Y, et al. Wellness programme at the workplace promotes dietary change and improves health indicators in a longitudinal retrospective study. Public Health Nutr. 2019;22(2):354-62.

122. Somers M, Birnbaum D, Casal J. Application of the person-centered model to stress and well-being research. Employee Relations: The International Journal. 2019;41(4):649-61.

123. Somerset S, Evans C, Blake H. Accessing Voluntary HIV Testing in the Construction Industry: A Qualitative Analysis of Employee Interviews from the Test@Work Study. Int J Environ Res Public Health. 2021;18(8).

124. Stephens SK, Eakin EG, Clark BK, Winkler EAH, Owen N, LaMontagne AD, et al. What strategies do desk-based workers choose to reduce sitting time and how well do they work? Findings from a cluster randomised controlled trial. Int J Behav Nutr Phys Act. 2018;15(1):98.

125. Stephenson A, Garcia-Constantino M, Murphy MH, McDonough SM, Nugent CD, Mair JL. The "Worktivity" mHealth intervention to reduce sedentary behaviour in the workplace: a feasibility cluster randomised controlled pilot study. BMC Public Health. 2021;21(1):1416.

126. Sun Y, Wang A, Yu S, Hagger MS, Chen X, Fong SSM, et al. A blended intervention to promote physical activity, health and work productivity among office employees using intervention mapping: a study protocol for a cluster-randomized controlled trial. BMC Public Health. 2020;20(1):994.

127. Tan AM, LaMontagne AD, English DR, Howard P. Efficacy of a workplace osteoporosis prevention intervention: a cluster randomized trial. BMC Public Health. 2016;16(1):859.

128. Tan AM, Lamontagne AD, Sarmugam R, Howard P. A cluster-randomised, controlled trial to assess the impact of a workplace osteoporosis prevention intervention on the dietary and physical activity behaviours of working women: study protocol. BMC Public Health. 2013;13:405.

129. Thogersen-Ntoumani C, Black J, Lindwall M, Whittaker A, Balanos GM. Presenteeism, stress resilience, and physical activity in older manual workers: a person-centred analysis. Eur J Ageing. 2017;14(4):385-96.

130. Thorndike AN, McCurley JL, Gelsomin ED, Anderson E, Chang Y, Porneala B, et al. Automated Behavioral Workplace Intervention to Prevent Weight Gain and Improve Diet: The ChooseWell 365 Randomized Clinical Trial. JAMA Netw Open. 2021;4(6):e2112528.

131. Tognetto A, Zorzoli E, Franco E, Gervasi G, Paglione L, Di Ninno F, et al. Seasonal influenza vaccination among health-care workers: the impact of different tailored programs in four University hospitals in Rome. Hum Vaccin Immunother. 2020;16(1):81-5.

132. Tshabalala AMET, Taylor M. An innovation to improve health outcomes in Amajuba district, KwaZulu-Natal, South Africa. Development Southern Africa. 2018;35(4):497-510.

133. Tucker S, Farrington M, Lanningham-Foster LM, Clark MK, Dawson C, Quinn GJ, et al. Worksite Physical Activity Intervention for Ambulatory Clinic Nursing Staff. Workplace Health Saf. 2016;64(7):313-25.

134. van de Ven D, Schuring M, Kouwenhoven-Pasmooij TA, Blom P, Burdorf A, Robroek SJW. Reach and effectiveness of a worksite health promotion program combining a preventive medical examination with motivational interviewing; a quasi-experimental study among workers in low socioeconomic position. BMC Public Health. 2023;23(1):2130.

135. van Doorn D, Richardson N, Meredith D, Blake C, McNamara J. Study protocol: Evaluation of the 'real-world' Farmers Have Hearts - Cardiovascular Health Program. Prev Med Rep. 2022;30:102010.

136. van Heijster H, van Berkel J, Bakker M, Boot C, de Vet E. Process evaluation of workplace health promotion in a sheltered workplace: a care ethics perspective. Health Promot Int. 2023;38(2).

137. van Holland BJ, Reneman MF, Soer R, Brouwer S, de Boer MR. Effectiveness and Cost-benefit Evaluation of a Comprehensive Workers' Health Surveillance Program for Sustainable Employability of Meat Processing Workers. J Occup Rehabil. 2018;28(1):107-20.

138. van Holland BJ, de Boer MR, Brouwer S, Soer R, Reneman MF. Sustained employability of workers in a production environment: design of a stepped wedge trial to evaluate effectiveness and cost-benefit of the POSE program. BMC Public Health. 2012;12:1003.

139. Varma P, Postnova S, Phillips AJK, Knock S, Howard ME, Rajaratnam SMW, et al. Pilot feasibility testing of biomathematical model recommendations for personalising sleep timing in shift workers. J Sleep Res. 2023:e14026.

140. Verburgh M, Verdonk P, Appelman Y, Brood-van Zanten M, Hulshof C, Nieuwenhuijsen K. Workplace Health Promotion Among Ethnically Diverse Women in Midlife With a Low Socioeconomic Position. Health Educ Behav. 2022;49(6):1042-55.

141. Viester L, Verhagen EA, Proper KI, van Dongen JM, Bongers PM, van der Beek AJ. VIP in construction: systematic development and evaluation of a multifaceted health programme aiming to improve physical activity levels and dietary patterns among construction workers. BMC Public Health. 2012;12:89.

142. Viester L, Verhagen E, Bongers PM, van der Beek AJ. Effectiveness of a Worksite Intervention for Male Construction Workers on Dietary and Physical Activity Behaviors, Body Mass Index, and Health Outcomes: Results of a Randomized Controlled Trial. Am J Health Promot. 2018;32(3):795-805.

143. von Lengerke T, Lutze B, Krauth C, Lange K, Stahmeyer JT, Chaberny IF. Promoting Hand Hygiene Compliance: PSYGIENE-a Cluster-Randomized Controlled Trial of Tailored Interventions. Dtsch Arztebl Int. 2017;114(3):29-36.

144. Walker N, Dobbing R. A redesigned training and staff support programme to enhance job retention in employees with moderate-severe depression. Mental Health and Social Inclusion. 2021;25(3):279-95.

145. Wallis A, Robertson J, Bloore RA, Jose PE. Differences and similarities between leaders and nonleaders on psychological distress, well-being, and challenges at work. Consulting Psychology Journal: Practice and Research. 2021;73(4):325-48.

146. Walthouwer MJL, Oenema A, Soetens K, Lechner L, de Vries H. Implementation of web-based interventions by Dutch occupational health centers. Health Promot Int. 2017;32(5):818-30.

147. Watkins C, Macy G, Golla V, Lartey G, Basham J. The "Total Worker Health" Concept: A Case Study in a Rural Workplace. J Occup Environ Med. 2018;60(5):387-91.

148. Wollesen B, Hagemann D, Pabst K, Schluter R, Bischoff LL, Otto AK, et al. Identifying Individual Stressors in Geriatric Nursing Staff-A Cross-Sectional Study. Int J Environ Res Public Health. 2019;16(19).

149. Yan L, Li J, Lin P, Sun J. Psychological Stress and Coping Strategy Profiles Among Frontline Medical Workers During the COVID-19 Pandemic. J Nerv Ment Dis. 2023;211(12):954-60.

150. Yao J, Steinberg D, Turner EL, Cai GY, Cameron JR, Hybels CF, et al. When Shepherds Shed: Trajectories of Weight-Related Behaviors in a Holistic Health Intervention Tailored for US Christian Clergy. J Relig Health. 2024;63(3):1849-66.

151. Zhai D, Schiavone G, Van Diest I, Vrieze E, DeRaedt W, Van Hoof C. Ambulatory Smoking Habits Investigation based on Physiology and Context (ASSIST) using wearable sensors and mobile phones: protocol for an observational study. BMJ Open. 2019;9(9):e028284.

152. Zhang E, Dai Z, Wang C, Hu J, Wang S, Zhang L, et al. Targeting COVID-19 vaccine hesitancy among nurses in Shanghai: A latent profile analysis. Frontiers in Public Health. 2022;10.

153. Zhang Y, Cordina-Duverger E, Komarzynski S, Attari AM, Huang Q, Aristizabal G, et al. Digital circadian and sleep health in individual hospital shift workers: A cross sectional telemonitoring study. EBioMedicine. 2022;81:104121.

154. Zoellner J, You W, Almeida F, Blackman KC, Harden S, Glasgow RE, et al. The Influence of Health Literacy on Reach, Retention, and Success in a Worksite Weight Loss Program. Am J Health Promot. 2016;30(4):279-82.
